# Endothelial-derived FABP4 constitutes the majority of basal circulating hormone and regulates lipolysis-driven insulin secretion

**DOI:** 10.1172/jci.insight.164642

**Published:** 2023-07-24

**Authors:** Karen E. Inouye, Kacey J. Prentice, Alexandra Lee, Zeqiu B. Wang, Carla Dominguez-Gonzalez, Mu Xian Chen, Jillian K. Riveros, M. Furkan Burak, Grace Y. Lee, Gökhan S. Hotamışlıgil

**Affiliations:** 1 Sabri Ülker Center for Metabolic Research, Harvard T.H. Chan School of Public Health, Department of Molecular Metabolism, Boston, Massachusetts, USA.; 2 Broad Institute of Harvard and MIT, Cambridge, Massachusetts, USA.

**Keywords:** Endocrinology, Metabolism, Adipose tissue, Endothelial cells, Insulin

## Abstract

Fatty acid binding protein 4 (FABP4) is a lipid chaperone secreted from adipocytes upon stimulation of lipolysis. Circulating FABP4 levels strongly correlate with obesity and metabolic pathologies in experimental models and humans. While adipocytes have been presumed to be the major source of hormonal FABP4, this question has not been addressed definitively in vivo. We generated mice with *Fabp4* deletion in cells known to express the gene — adipocytes (Adipo-KO), endothelial cells (Endo-KO), myeloid cells (Myeloid-KO), and the whole body (Total-KO) — to examine the contribution of these cell types to basal and stimulated plasma FABP4 levels. Unexpectedly, baseline plasma FABP4 was not significantly reduced in Adipo-KO mice, whereas Endo-KO mice showed ~87% reduction versus WT controls. In contrast, Adipo-KO mice exhibited ~62% decreased induction of FABP4 responses to lipolysis, while Endo-KO mice showed only mildly decreased induction, indicating that adipocytes are the main source of increases in FABP4 during lipolysis. We did not detect any myeloid contribution to circulating FABP4. Surprisingly, despite the nearly intact induction of FABP4, Endo-KO mice showed blunted lipolysis-induced insulin secretion, identical to Total-KO mice. We conclude that the endothelium is the major source of baseline hormonal FABP4 and is required for the insulin response to lipolysis.

## Graphical abstract

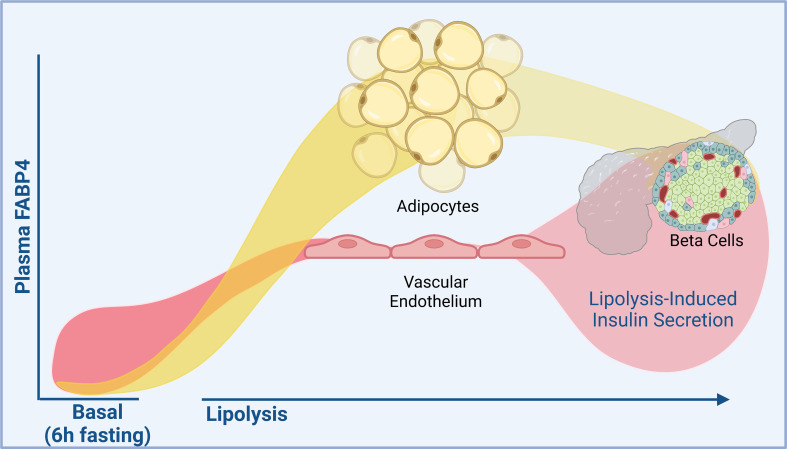

## Introduction

Fatty acid binding protein-4 (FABP4, also known as aP2) is a 15 kDa protein belonging to a highly conserved family of lipid binding proteins, each with specific tissue distributions (1). FABP4 was first identified as the adipocyte-specific isoform, regulated during differentiation, and is one of the most abundant proteins in mature adipocytes, comprising up to 6% of total adipocyte cytosolic protein content (2). Total genetic deletion of *Fabp4* in mice confers protection against obesity-associated insulin resistance, glucose intolerance, inflammation, atherosclerosis, and certain cancers (3–7). Importantly, multiple independent studies in humans carrying a low-expression variant of *FABP4* demonstrated lower incidence of type 2 diabetes, decreased circulating triglycerides and cholesterol, and reduced incidence of cardiometabolic disease (8, 9).

FABP4 has been generally considered as a cytosolic protein that exerts its actions as an intracellular protein. Similar to several other FABP isoforms, it has been detected in serum (10). While the presence of other circulating isoforms has been ascribed to cell death and leakage from the degenerating source cells (11–12), we have demonstrated that FABP4 is, in fact, secreted from adipocytes in a regulated manner, through a nonclassical pathway, induced by lipolysis (13, 14). Currently, it is the only known adipocyte hormonal signal accompanying the breakdown of energy stores during lipolysis. Subsequently, it was shown that a major proportion of FABP4 secretion from adipocytes occurs through a lysosomal pathway, a mechanism extremely rare for mammalian protein secretion (15). Circulating FABP4 levels correlate positively with BMI and indicators of metabolic syndrome in humans, and they are increased in mice with dietary or genetic obesity (10, 13, 16, 17), raising the possibility that excessive adipose tissue release of FABP4 may, in fact, be the strong contributor to metabolic dysregulation seen in mouse models and humans (18). Taken together, these observations raise interest in antibody-based targeting of FABP4 hormone for therapeutic purposes, and proof of principle studies in experimental models demonstrate the efficacy and feasibility of this approach (19, 20).

FABP4 secretion by cultured adipocytes and in vivo is inducible, stimulated by prolonged fasting and lipolytic signals (13, 14). Induction of lipolysis in mice with β-adrenergic agonists potently stimulates FABP4 secretion (13). It has been shown that acute β-adrenergic stimulation of lipolysis in vivo is associated with a rapid, robust, and transient induction of insulin secretion (21, 22). The mechanisms and functional consequences of this rather unusual link have remained enigmatic. Interestingly, studies in FABP4-deficient animals demonstrated that the presence of FABP4 is an important requirement for this lipolysis-driven insulin secretion, as insulin responses to lipolysis are blunted by at least 60% in lean *Fabp4^–/–^* mice, whereas they remain intact in response to other signals (23). Most critical to note is that glucose-stimulated insulin secretion (GSIS) from islets of FABP4-deficient animals is enhanced, and these mice are strongly protected against both type 1 and type 2 diabetes (3, 5, 24). This altered regulation of insulin secretion is the most striking phenotype we have observed in lean *Fabp4^–/–^* mice, as they do not show changes in other metabolic parameters such as body weight, glucose tolerance, and insulin sensitivity (3). Since *Fabp4^–/–^* mice are also protected against obesity-associated hyperinsulinemia, the decrease in lipolysis-driven insulin secretion has been considered as one potential contributing factor to their reduced hyperinsulinemia and improved metabolic health (3, 5, 23).

While FABP4 is most highly expressed in adipose tissue, it is also expressed in other cell types such as macrophages and endothelial cells (4, 25, 26). FABP4 is widely expressed in the endothelial cells of the microvasculature, with immunoreactivity detected in capillaries and small veins of the heart, lung, kidney, liver, muscle, and pancreas (25, 26), but little is known about the physiological role of endothelial FABP4. Endothelial FABP4 may play a role in blood vessel growth, as absence of FABP4 in endothelial cells reduces proliferation and tumor angiogenesis (26, 27). Endothelial FABP4 may also play a role in fatty acid transport into tissues, as shown in cultured cells (28), and in vivo in genetic-deficiency models (25). There is increasing evidence that the endothelium has a regulatory role in metabolism and that endothelial dysfunction contributes to obesity-related pathologies (29, 30). Given the presence of FABP4 in endothelial cells, it is important to understand the FABP4 secretory capacity of the endothelium and the physiological and pathophysiological roles of endothelial FABP4 to guide potential translational efforts and strategies.

To investigate the contribution of adipocytes, endothelial cells, and macrophages to the circulating FABP4 pool and the physiological roles of these sources of FABP4, we generated a mouse model with deletion of FABP4 in adipocytes, endothelial cells, myeloid cells, or the whole body. As our first investigation into the roles of FABP4 from these cell types, we examined FABP4 levels in lean mice under basal conditions and in response to lipolysis. Surprisingly, we found that ~87% of baseline circulating FABP4 in lean mice is derived from endothelial cells, whereas only ~11% is contributed by adipocytes. In contrast, during lipolysis, adipocytes were the main source of increases in circulating FABP4. Unexpectedly, despite near-normal induction of FABP4 responses to lipolysis, mice lacking endothelial FABP4 exhibited reduced lipolysis-induced insulin responses to the same degree as whole-body *Fabp4^–/–^* mice. This was in contrast to enhanced GSIS from isolated islets of endothelial FABP4-deletion mice. In these studies, we did not observe any contribution of myeloid cells to either baseline or stimulated circulating FABP4 levels. These data indicate that endothelial cells are the major contributors to baseline circulating FABP4 and that endothelial-source FABP4 is critical for pancreatic β cell insulin responses to lipolysis.

## Results

### Development and validation of a tissue-specific FABP4 deletion mouse model.

To study the physiological role of different tissue sources of FABP4, we developed a humanized *Fabp4*-floxed mouse model. We crossed *Fabp4*-floxed mice to Adiponectin-Cre, Tek-Cre, and CMV-Cre mice to generate mice with deletion of FABP4 in adipocytes (Adipo-KO), endothelial cells (Endo-KO), and the whole body (Total-KO), respectively (Supplemental Figure 1A; supplemental material available online with this article; https://doi.org/10.1172/jci.insight.164642DS1). Since Tek-Cre mouse models can also delete from some hematopoietic populations (31), and we have confirmed these observations in peritoneal macrophages (Supplemental Figure 2), we also crossed *Fabp4*-floxed mice with LyzM-Cre mice to delete FABP4 from the myeloid population (Myeloid-KO) in order to further differentiate the potential contributions of endothelial and macrophage FABP4.

**Figure 1:**
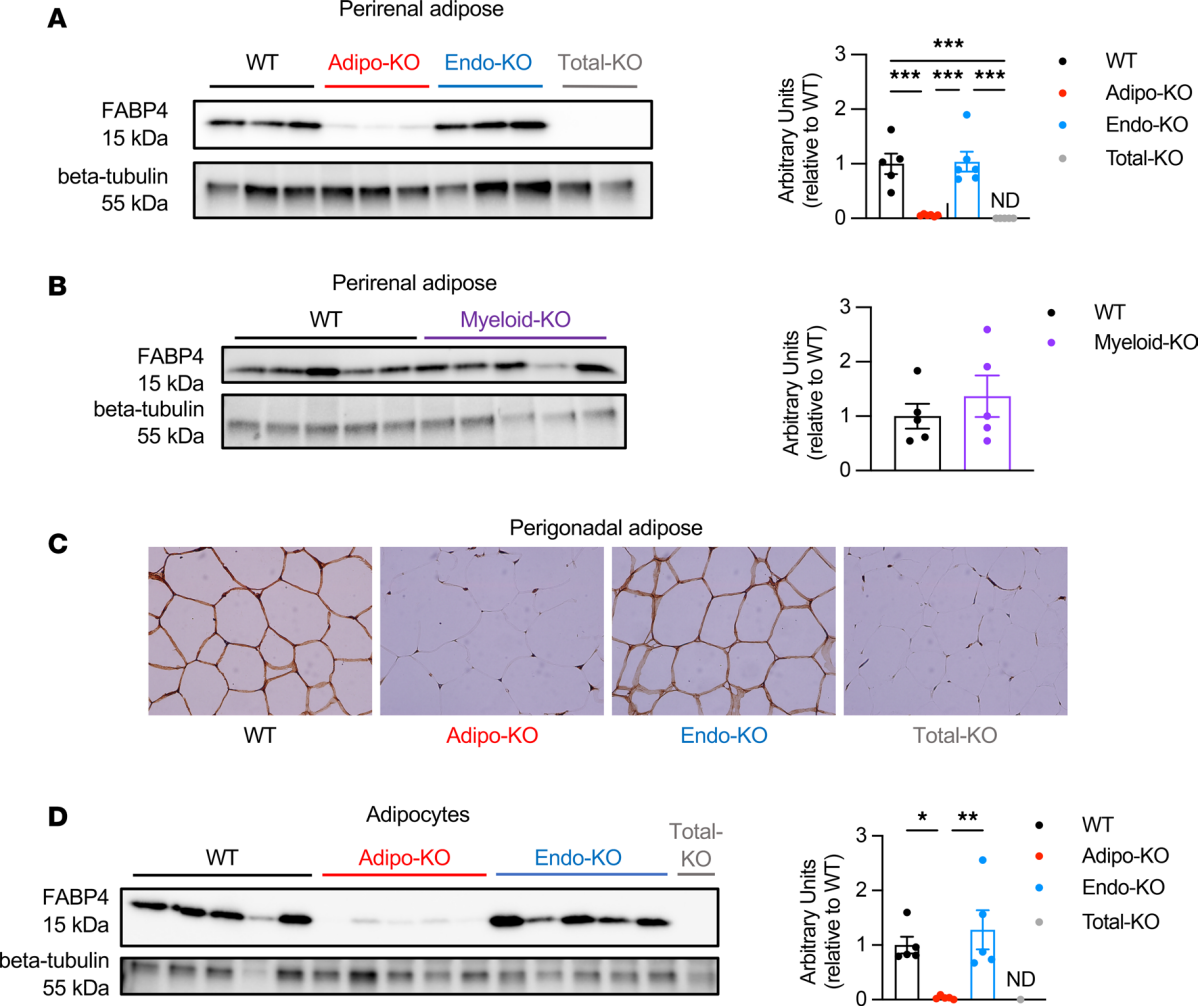
Validation of tissue-specific deletion of FABP4 from adipocytes. (**A**) Representative immunoblots (*n* = 2) and quantification of FABP4 protein relative to β-tubulin loading control in perirenal adipose tissue of WT, Adipo-KO, Endo-KO, and Total-KO mice. WT, Total-KO: *n* = 5/group; Adipo-KO, Endo-KO: *n* = 6/group. (**B**) Immunoblots and quantification of FABP4 protein relative to β-tubulin loading control in perirenal adipose tissue of WT and Myeloid-KO mice. *n* = 5/group. (**C**) Representative FABP4 immunostaining in perigonadal adipose tissue from WT, Adipo-KO, Endo-KO, and Total-KO mice. Magnification, 400***×***. (**D**) Immunoblots and quantification of FABP4 protein relative to β-tubulin loading control in isolated perigonadal adipocytes of WT, Adipo-KO, Endo-KO, and Total-KO mice. WT, Adipo-KO, Endo-KO: *n* = 5/group; Total-KO: *n* = 1. *P < 0.05, **P < 0.01, ***P < 0.001 by 1-way ANOVA, followed by Tukey’s multiple-comparison test (**A** and **D**), or by t test (**B**). ND, no signal detected.

We next validated the deletion of FABP4 in the specific tissues. In Western blots of perirenal, mesenteric, and brown adipose tissue (BAT), we observed near complete deletion of FABP4 protein in Adipo-KO mice (Figure 1A and Supplemental Figure 3, A and B; see complete unedited blots in the supplemental material). In contrast, Endo-KO and Myeloid-KO perirenal and mesenteric adipose tissue showed no differences in FABP4 protein expression from WT controls (Figure 1, A and B, Supplemental Figure 3A, and Supplemental Figure 4A). In BAT, Endo-KO mice showed a trend toward decreased FABP4 protein, and Myeloid-KO showed significantly reduced FABP4 compared with WT (Supplemental Figure 3B and Supplemental Figure 4B), suggesting some contribution of myeloid cells to BAT FABP4 at the whole-tissue level. Immunostaining of perigonadal adipose tissue (PGWAT) showed near absence of FABP4 in adipocytes of Adipo-KO but not Endo-KO mice (Figure 1C). In BAT, Adipo-KO showed a residual FABP4 signal in what appeared to be a nonadipocyte cell population (Supplemental Figure 3C), which is likely myeloid cells, given the decreased FABP4 protein in Myeloid-KO BAT. It should be noted that Total-KO PGWAT and BAT also showed some residual signal in the immunostaining. Since adipose from *Fabp4/5*^–/–^ mice, which we used as a negative control to validate this antibody, showed no signal at all (Supplemental Figure 3D), we surmise that this signal represents some cross-reactivity with FABP5, a closely related isoform to FABP4. We observed compensatory increases in FABP5 in perirenal adipose of Total-KO and Adipo-KO mice compared with WT (Supplemental Figure 5). Therefore, some of the signal in Adipo-KO adipose tissue could have also been due to cross-reactivity with FABP5. To verify specific deletion of FABP4 in adipocytes of Adipo-KO mice we fractionated the PGWAT depot and performed Western blots using protein from isolated adipocytes. As in whole adipose, isolated adipocytes of Adipo-KO mice had near complete deletion of FABP4 protein, whereas Endo-KO adipocytes did not differ in FABP4 protein expression from WT (Figure 1D).

**Figure 2:**
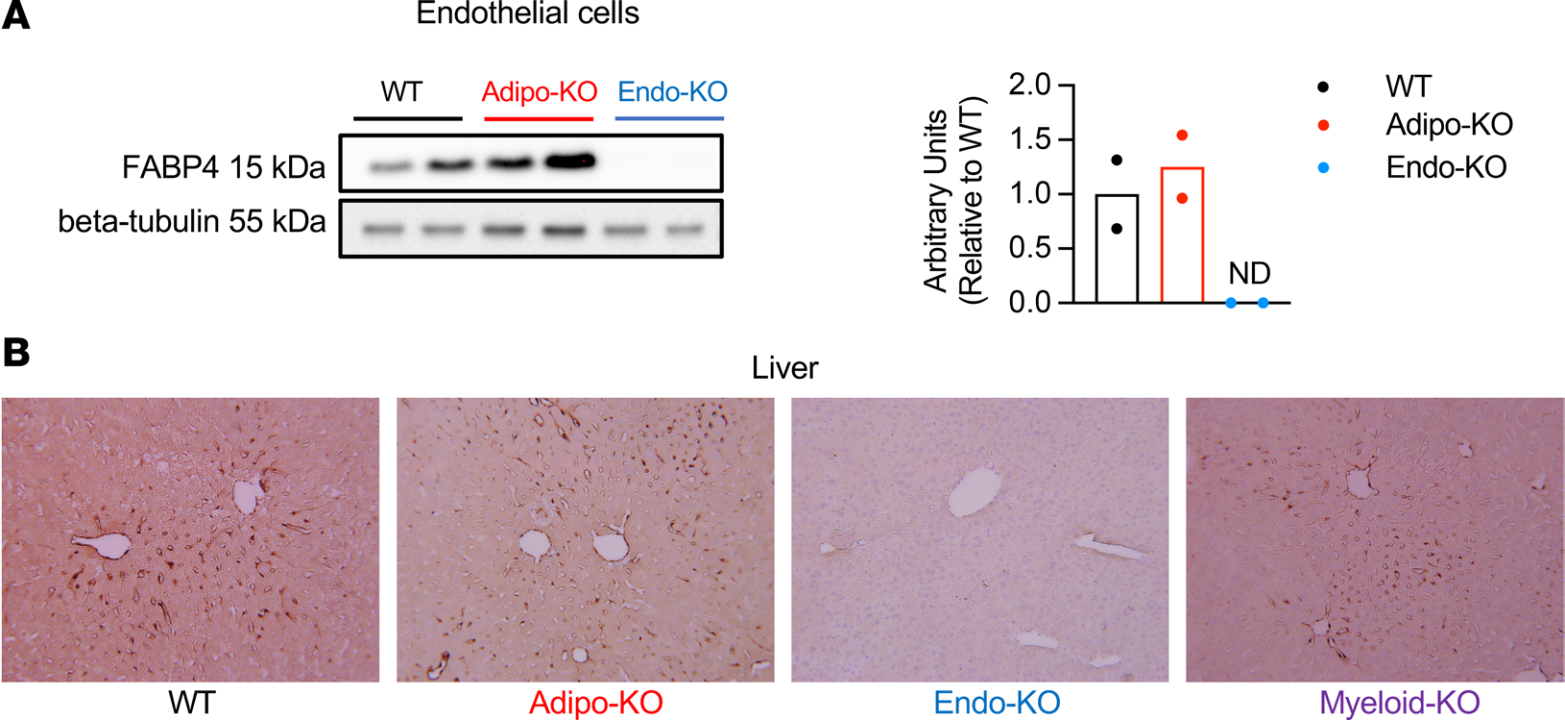
Validation of tissue-specific deletion of FABP4 from endothelial cells. (**A**) Immunoblots and quantification of FABP4 protein relative to β-tubulin loading control in CD31-isolated endothelial cells from liver, spleen, heart, and lungs of WT, Adipo-KO, and Endo-KO mice. *n* = 2/group. (**B**) Representative FABP4 immunostaining in livers of WT, Adipo-KO, Endo-KO, and Myeloid-KO mice; magnification, 200×. ND, no signal detected.

We next isolated endothelial cells from collagenase-digested liver alone or liver, spleen, heart, and lung using magnetic beads coated with CD31 antibody and performed immunoblots for FABP4 protein. Since CD31 is also expressed on Kupffer cells, we evaluated the purity of WT endothelial cells isolated with CD31 antibody by costaining with antibodies against CD31 and the macrophage marker F4/80, and we performed FACS analysis to determine the percentage of endothelial cells versus macrophages (Supplemental Figure 6A). Of the total pool of cells, 88.5% were positive for CD31 alone and 9.4% were costained with anti-CD31 and anti-F4/80, indicating that 88.5% of the cells were endothelial cells and 9.4% were macrophages. Isolated CD31^+^ cells from Endo-KO liver, spleen, heart, and lung showed no FABP4 signal in Western blots, whereas Adipo-KO endothelial cells showed similar FABP4 expression to WT (Figure 2A). Immunostaining of livers of WT mice showed FABP4 positivity in cells lining blood vessels and sinusoids, indicating FABP4 expression in vascular and sinusoidal endothelial cells or Kupffer cells (Figure 2B). Adipo-KO mice showed similar FABP4 staining patterns to WT, whereas Endo-KO mice showed no FABP4 signal in the liver. Western blots of whole liver also showed absence of FABP4 in Endo-KO livers, similar to Total-KO mice (Supplemental Figure 6B). Myeloid-KO mice showed FABP4 staining similar to WT mice (Figure 2B), supporting that the absence of FABP4 signal in Endo-KO mice was mainly due to deletion of FABP4 specifically in endothelial cells.

### Endothelial cells contribute ~87% of basal circulating FABP4 in lean mice.

**Figure 3:**
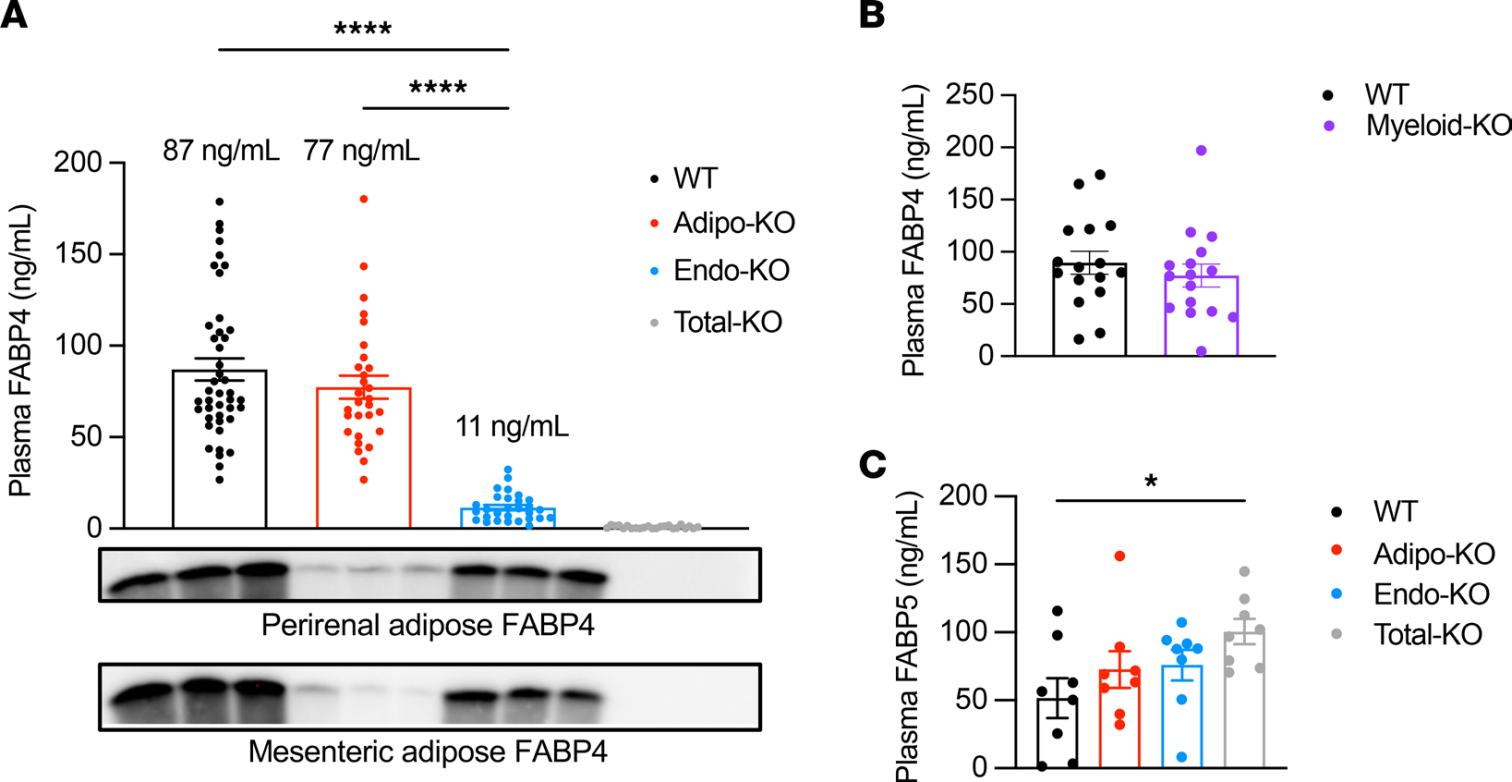
Endothelial cells contribute to approximately 87% of basal circulating FABP4 during 6-hour daytime fasting. (**A**) Plasma FABP4 levels in lean WT (*n* = 43), Adipo-KO (*n* = 29), Endo-KO (*n* = 29), and Total-KO (*n* = 23) male mice. Data are pooled from 5 experiments. Average age of mice is 13 weeks old. Immunoblots of perirenal and mesenteric adipose FABP4 protein expression, for comparison with plasma levels. (**B**) Plasma FABP4 levels in WT versus Myeloid-KO mice. Data are pooled from 2 experiments, *n* = 8/group/experiment. (**C**) Plasma FABP5 levels in WT, Adipo-KO, Endo-KO, and Total-KO mice. *n* = 8/group. All samples were collected from male mice after 6-hour daytime food withdrawal. *P < 0.05, ****P < 0.0001 by unpaired *t* test (**B**) or by 1-way ANOVA, followed by Tukey’s multiple-comparison test (**A** and **C**).

Although FABP4 is expressed in endothelial cells of several tissues and organs (25, 26), it is not known whether these cells contribute to circulating FABP4. To examine this question, we measured circulating FABP4 levels in lean WT, Adipo-KO, and Endo-KO mice. The data shown are a pool of FABP4 levels from multiple experiments in which blood was collected from lean male mice following 6-hour daytime food withdrawal. We chose 6-hour food withdrawal as a basal state in which metabolite and hormone levels are not influenced by recent food consumption, and catabolic pathways such as lipolysis are not engaged. Surprisingly, Adipo-KO mice only showed a nonsignificant ~11% reduction in baseline circulating FABP4 levels compared with WT controls (Figure 3A). In contrast, Endo-KO mice showed a striking ~87% decrease in basal plasma FABP4 concentrations. We observed comparable FABP4 levels in age-matched female mice, despite lower body weights (Supplemental Figure 7, A–C). Myeloid-KO mice did not show any changes in circulating FABP4 (Figure 3B), indicating that these cells do not contribute to FABP4 hormone at measurable levels and that the reduction in Endo-KO mice was indeed due to absence of FABP4 in endothelial cells. These data are in line with our previous observations in FABP4-deficient mice transplanted with WT BM (or WT mice receiving FABP4-deficient BM), where no myeloid contribution to circulating FABP4 was evident (13). Taken together, these data indicate that, in lean mice under baseline conditions, the majority of circulating FABP4 is derived from endothelial cells, rather than adipocytes.

In *Fabp4^–/–^* mice, plasma FABP5 levels are nearly doubled compared with WT controls (13). We observed increased plasma FABP5 only in Total-KO mice compared with WT (Figure 3C). Baseline 6-hour fasting glucose and insulin levels did not differ among WT, Adipo-KO, Endo-KO, and Total-KO mice matched for body weight (Table 1). HOMA1-IR values also did not differ between the groups, suggesting no differences in insulin sensitivity. These findings were not unexpected, given that we previously observed no differences in these parameters between lean WT and whole-body *Fabp4^–/–^* mice (3).

### Lipolysis-driven FABP4 secretion is derived mainly from adipocytes.

**Figure 4:**
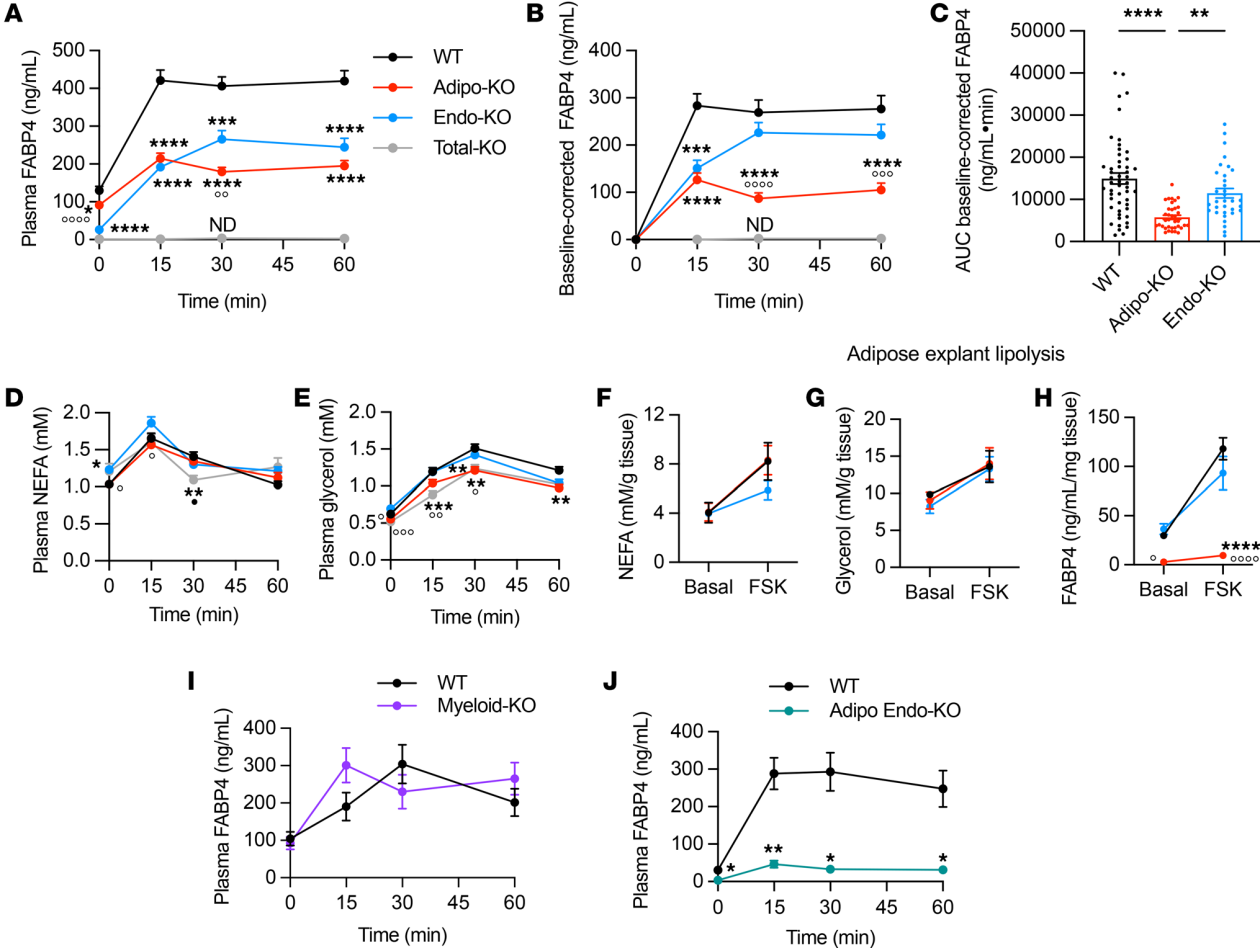
Lipolysis-driven FABP4 secretion is primarily from adipocytes. (**A**–**E**) Plasma FABP4 levels, baseline-corrected plasma FABP4, AUC of baseline-corrected plasma FABP4, nonesterified fatty acid (NEFA), and glycerol responses to 10 mg/kg isoproterenol-induced lipolysis in ~13-week-old WT (*n* = 54), Adipo-KO (*n* = 40), Endo-KO (*n* = 34), and Total-KO (*n* = 8 for FABP4, *n* = 15 for NEFA, glycerol) mice. Data for **A**–**E** are pooled from 6 experiments. (**F**–**H**) NEFA, glycerol, and FABP4 responses to FSK-induced lipolysis in perigonadal adipose explants from WT, Adipo-KO, and Endo-KO mice; *n* = 4/group. Data are normalized to amount of adipose tissue per culture well. (**I**) Plasma FABP4 responses to 10 mg/kg isoproterenol-induced lipolysis in WT and Myeloid-KO mice; *n* = 8/group. (**J**) WT versus Adipo Endo-KO mice with deletion of FABP4 in both adipocytes and endothelial cells; *n* = 6/group. All experiments were in male mice. ****P < 0.0001, ***P < 0.001, ** *P* <0.01, *P < 0.05 versus WT; °°°°*P* < 0.0001, °°°*P* < 0.001, °°*P* < 0.01, °*P* < 0.05 versus Endo-KO; •*P* < 0.05 versus Adipo-KO, by mixed-effects analysis followed by Tukey’s multiple-comparison test (**A**, **B**, **D**, and **E**), or 2-way ANOVA followed by Tukey’s (**F**, **G**, and **H**) or Sidak’s (**I** and **J**) multiple-comparison test. ****P < 0.0001, ** *P* <0.01 by 1-way ANOVA followed by Tukey’s multiple-comparison test (**C**). ND, no signal detected.

We have previously shown that lipolysis is a key driver of FABP4 secretion in cultured adipocytes, adipose tissue explants, and in whole animals (13, 14). To determine the cellular source of these increases in FABP4 in vivo, we compared FABP4 levels in circulation in WT, Adipo-KO, and Endo-KO mice following isoproterenol-induced lipolysis. Despite no significant reductions in basal plasma FABP4 levels, male Adipo-KO mice showed significantly diminished FABP4 secretory responses to isoproterenol compared with WT controls, which was particularly evident when corrected for baseline FABP4 levels (Figure 4, A and B). This corresponded to a 62% decrease in the AUC of the baseline-corrected Adipo-KO response (Figure 4C). Endo-KO mice also showed decreased absolute FABP4 levels that were only slightly higher than Adipo-KO mice. However, correction for baseline FABP4 revealed that Endo-KO mice showed induction of FABP4 secretion that was much closer to WT mice. A similar pattern of FABP4 response to isoproterenol was observed in female mice, though levels were lower than males (Supplemental Figure 7, D and E). Nonesterified fatty acid (NEFA) responses to induction of lipolysis were decreased at 30 minutes in Total-KO mice compared with WT (Figure 4D). Glycerol responses were also mildly decreased in Total-KO and Adipo-KO mice versus WT controls and Endo-KO mice (Figure 4E). These data are in line with previous observations in whole-body *Fabp4^–/–^* mice (23, 32) and show that absence of adipocyte FABP4 may underlie this phenotype.

Notably, adipocyte FABP4 deletion did not completely abrogate the FABP4 response to isoproterenol, and likewise, Endo-KO mice still had a 23% reduction in the AUC of the baseline-corrected FABP4 response. To rule out whether the small induction of FABP4 in Adipo-KO mice may have been due to residual adipose secretion of FABP4, due to incomplete deletion in adipocytes, we performed forskolin-induced (FSK-induced) lipolysis in adipose explants from WT, Adipo-KO, and Endo-KO mice. There was no effect of adipose or endothelial deletion of FABP4 on explant NEFA and glycerol responses to FSK (Figure 4, F and G). Induction of lipolysis in explants led to robust and similar increases in FABP4 from WT and Endo-KO explants (Figure 4H). Importantly, Adipo-KO explants showed essentially no baseline or FSK-induced FABP4 response. These data suggest that the majority of the FABP4 secretion observed in Adipo-KO mice in vivo may in fact be derived from the endothelial cell population.

Since Tek-Cre is also capable of deletion from myeloid cells, we also performed lipolysis in WT versus Myeloid-KO mice. FABP4 responses in Myeloid-KO mice were identical to WT controls (Figure 4I), indicating that the mildly reduced FABP4 response in Endo-KO mice was indeed due to absence of endothelial and not macrophage FABP4. We next determined whether endothelial cells contributed to the small induction of FABP4 observed in Adipo-KO mice. To do this, we crossed Adipo-KO mice with Endo-KO mice to delete FABP4 in both cell types. Mice with deletion in both depots had nearly 90% reduction in FABP4 responses to lipolysis (Figure 4J), indicating that the residual induction of FABP4 secretion in Adipo-KO mice was primarily from endothelial cells. Since we did not observe any alteration in endothelial cell FABP4 in Adipo-KO mice (Figure 2A), the endothelial contribution to lipolysis-induced circulating FABP4 levels is not the result of a compensatory increase in endothelial FABP4 expression. Taken together, our data show that adipocyte FABP4 secretion is potently induced in response to lipolytic stimulation; adipocytes contribute to at least 62% of the increase in FABP4, with a much smaller contribution from the endothelial pool. Notably, due to the high contribution of endothelial cells to baseline plasma FABP4, endothelial-source FABP4 is required for maximal levels during lipolysis.

### Regulation of endothelial FABP4 secretion differs from adipocytes.

**Figure 5:**
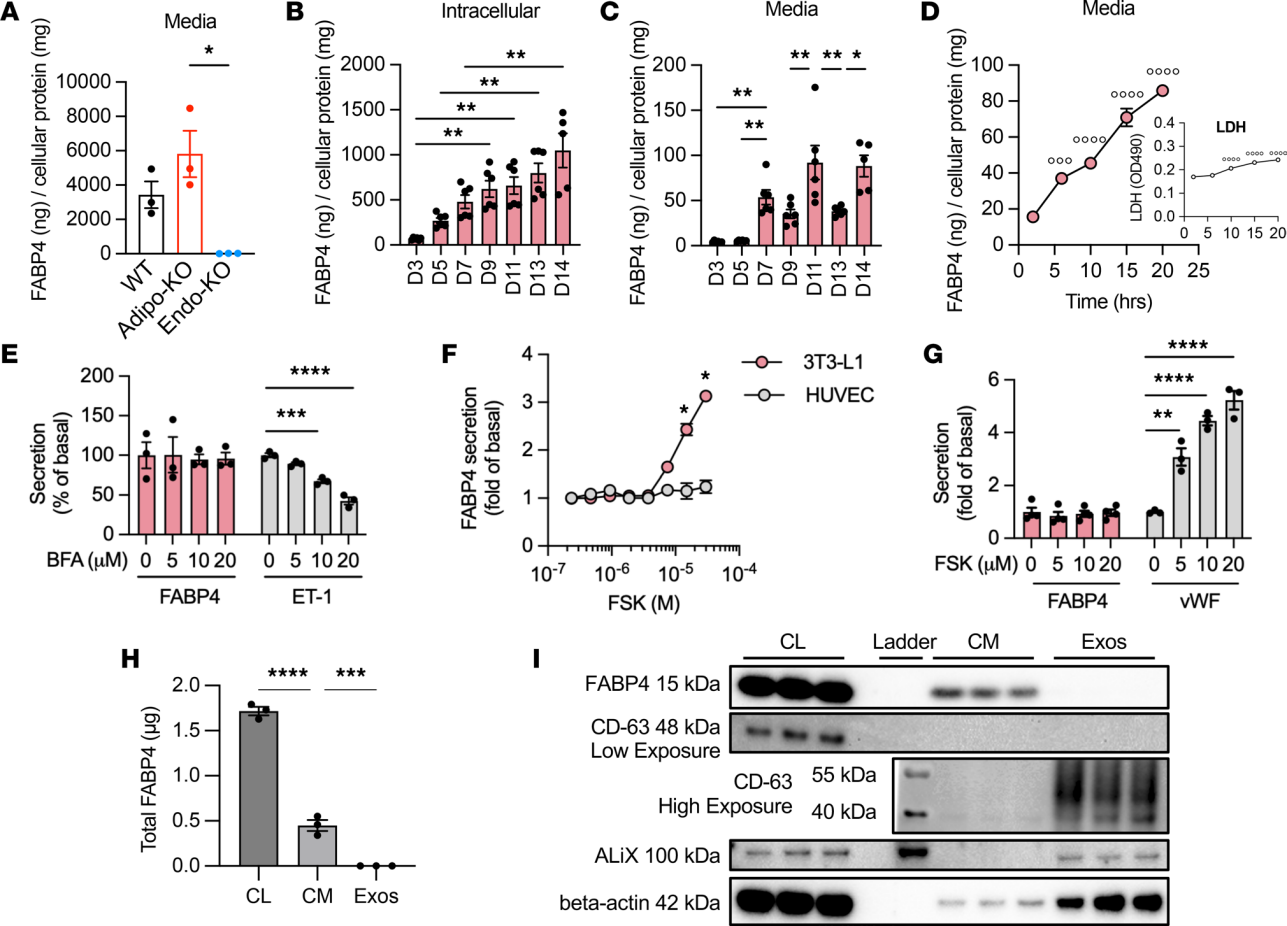
Endothelial and adipocyte FABP4 secretion are differentially regulated. (**A**) Twelve-hour conditioned media FABP4 levels from CD31-isolated endothelial cells from liver, heart, and lungs of WT, Adipo-KO, and Endo-KO mice, normalized to total cellular protein; *n* = 3 mice/group. (**B** and **C**) FABP4 levels in HUVEC lysates and 5-hour conditioned media, normalized to total cellular protein at days 3 through 14 after seeding. Pool of 2 experiments; *n* = 6/time point. (**D**) Time-course of cumulative FABP4 levels in media of day 7 HUVECs; *n* = 4/time point. Inset: Media lactate dehydrogenase (LDH) levels during the same time course. °°°°*P* < 0.0001, °°°*P* < 0.001 versus 0 hours by 1-way ANOVA followed by Dunnett’s multiple-comparison test. (**E**) Effects of the ER-Golgi pathway inhibitor, brefeldin A (BFA), on FABP4 and endothelin-1 (ET-1) secretion from day 11 HUVECs; *n* = 3/BFA dose. (**F**) Effects of forskolin (FSK) on FABP4 secretion in HUVECs versus 3T3-L1 adipocytes. *n* = 3/FSK dose. *P < 0.05 versus HUVEC by 2-way ANOVA followed by Sidak’s multiple-comparison test. (**G**) Effects of FSK on FABP4 and von Willebrand Factor (vWF) secretion from day 11 HUVECs. FABP4, *n* = 4/FSK dose; vWF, *n* = 3/FSK dose. (**H**) Total FABP4 measured by ELISA in HUVEC cell lysates (CL), conditioned media (CM), and exosomes (Exos) isolated from CM; *n* = 3. (**I**) Western blots of FABP4, exosome markers CD-63 and ALiX, and double-membrane protein β-actin in HUVEC cell lysates, conditioned media, and exosomes. *P < 0.05, **P < 0.01, ***P < 0.001, ****P < 0.0001 by 1-way ANOVA followed by Sidak’s (**A**), Tukey’s (**B**, **C**, and **H**), or Dunnett’s (**E** and **G**) multiple-comparison test.

To examine the FABP4 secretory capacity of endothelial cells, we cultured isolated endothelial cells (using the CD31 affinity method described above) pooled from liver, heart, and lungs of WT, Adipo-KO, and Endo-KO mice and collected conditioned media for assessment of FABP4 levels. WT and Adipo-KO endothelial cells both secreted FABP4 in similar amounts, providing direct evidence that endothelial cells secrete FABP4 (Figure 5A), which is lost, as expected, in Endo-KO cells. There was no compensatory upregulation of FABP5 in Endo-KO mice, as FABP5 levels in Endo-KO endothelial cell conditioned media and lysates did not differ from WT (Supplemental Figure 8, A and B).

We also performed studies in human umbilical vein endothelial cells (HUVECs) to investigate the regulation of endothelial FABP4 secretion. We first tested FABP4 expression at different stages of HUVEC culture, as it is known that HUVECs grown to the tightly packed monolayer referred to as a “cobblestone” appearance more closely resemble the morphology and phenotype of endothelial cells in vivo (33). Protein levels of FABP4 in HUVECs were maximal at day 14 after seeding, after the cells had reached the cobblestone phenotype (Figure 5B and Supplemental Figure 9). HUVEC secretion of FABP4 into the media showed an increase beginning on day 7 (Figure 5C). While secretion of FABP4 by day 7 HUVECs coincided with a small increase in media lactate dehydrogenase (LDH) levels (Figure 5D), the magnitude of the increase in FABP4 secretion exceeded the increase in LDH, indicating that the increase in FABP4 is primarily due to secretion from HUVECs, rather than release due to cell death.

We next tested how endothelial FABP4 secretion compares with adipocytes. In day 7 HUVECs, cellular FABP4 levels were ~10-fold lower than differentiated 3T3-L1 adipocytes, and 2-hour conditioned media FABP4 levels were ~640-fold lower than in 3T3-L1 media (Table 2), indicating that endothelial cells have much lower FABP4 content and basal secretion than adipocytes. Adipocyte FABP4 is not secreted via the classical endoplasmic reticulum–Golgi (ER-Golgi) pathway (14, 15, 34) but is mostly released from secretory lysosomes (15). When we treated day 11 cobblestone HUVECs with the ER-Golgi pathway inhibitors, brefeldin A or monensin, FABP4 secretion was not inhibited by either agent (Figure 5E and Supplemental Table 1), indicating that, like adipocytes, endothelial FABP4 secretion does not occur through the classical ER-Golgi pathway. Secretion of endothelin-1, a protein released via the ER-Golgi pathway (35), was inhibited by brefeldin A (Figure 5E), indicating that the ER-Golgi secretory mechanism was functional in our HUVECs. We also tested the effect of the lysosomal secretion inhibitors chloroquine and ammonium chloride. Whereas chloroquine and ammonium chloride inhibit isoproterenol or FSK-induced FABP4 release by adipocytes (15), they did not inhibit HUVEC FABP4 secretion (Supplemental Table 1). Increased intracellular calcium also induces FABP4 release by human adipocytes (34), but this response was also not present in HUVECs when histamine was used to raise intracellular Ca^2+^ (Supplemental Table 1). Taken together, our data show that FABP4 secretion by endothelial cells is not stimulated by the same mechanisms as in adipocytes.

We next tested the effects of the lipolytic agents — FSK, IBMX, isoproterenol, and CL-316,243 — on HUVEC FABP4 secretion. FSK induced robust secretion of FABP4 by 3T3-L1 adipocytes but had no effect on HUVEC FABP4 secretion (Figure 5F). Similarly, IBMX, isoproterenol, and CL-316,243 did not induce HUVEC FABP4 secretion (Supplemental Table 1). This contrasts with the effect of isoproterenol to induce endothelial FABP4 secretion in vivo. The lack of responsiveness to FSK was not due to suboptimal growing conditions of the HUVEC cultures, as HUVEC secretion of von Willebrand Factor (vWF) responded robustly to FSK (Figure 5G). This difference between in vivo and in vitro responses to cAMP-mediated stimulation indicates that there may be additional factors required for endothelial FABP4 secretion in vivo or that FABP4 may be coming from a specialized endothelial subpopulation that is not represented in these culture systems.

### FABP4 is not secreted from HUVECs via exosomes.

We and others have shown that a small fraction of adipocyte FABP4 is released via exosomes (14, 36). To determine whether endothelial cells also secrete FABP4 via this vesicular pathway, we analyzed the conditioned media of cobblestone-stage HUVECs. HUVEC exosomes showed no detectable FABP4, despite the presence of FABP4 in HUVEC lysates and conditioned media (Figure 5H). We confirmed the purity of the exosome fraction by Western blot, using the exosomal markers CD35 and ALiX, and the double-membrane marker β-actin, and we showed that the exosome fraction expressed these markers but did not express FABP4 (Figure 5I).

### Lipolysis-driven insulin secretion is blunted in mice lacking endothelial FABP4.

**Figure 6:**
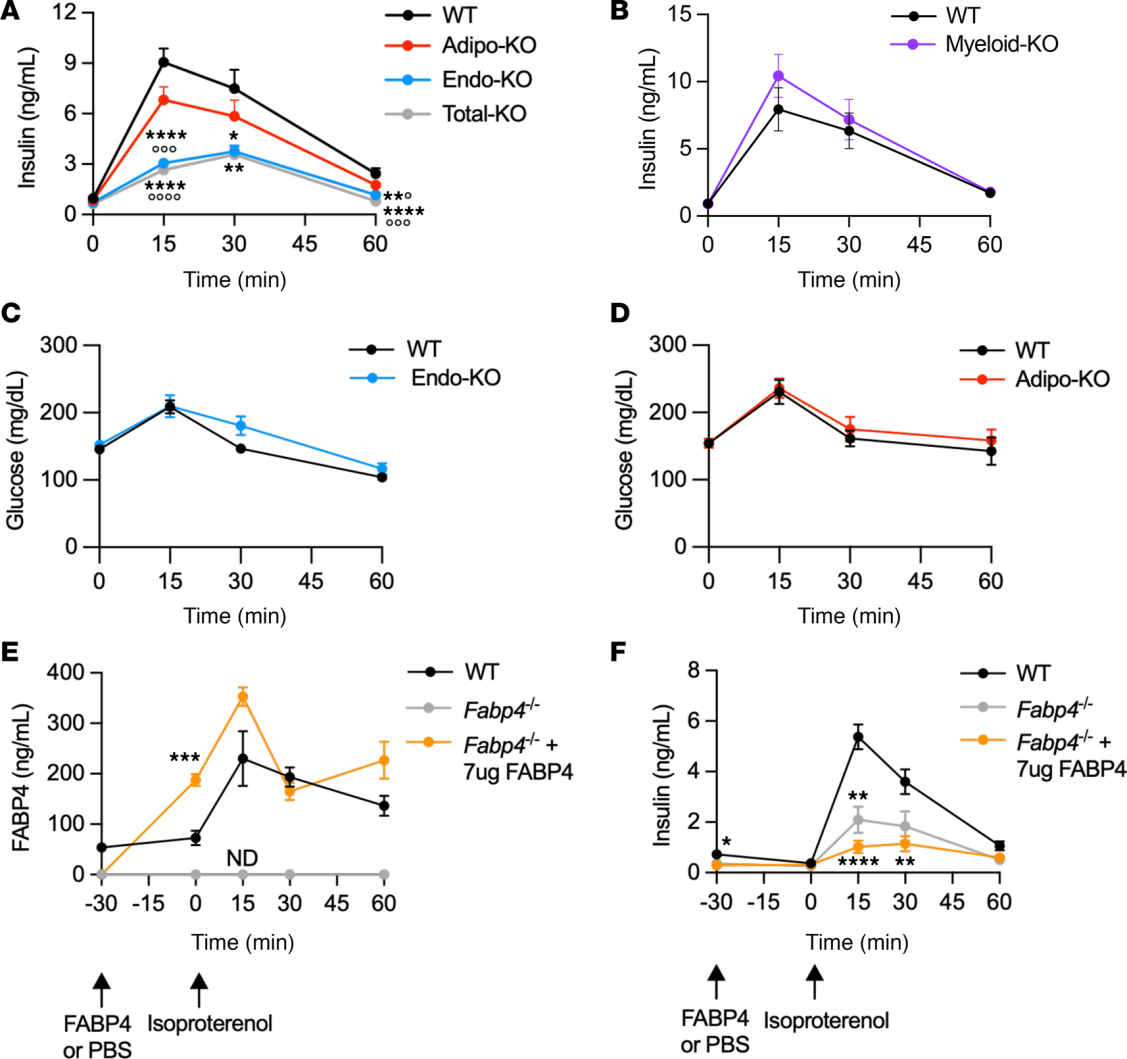
Lipolysis-driven insulin secretion is blunted in mice lacking endothelial but not adipocyte FABP4. (**A**) Plasma insulin responses to 10 mg/kg isoproterenol-induced lipolysis in WT (*n* = 52), Adipo-KO (*n* = 40), Endo-KO (*n* = 34), and Total-KO (*n* = 15) mice. Data are pooled from 6 experiments. (**B**) Plasma insulin responses to 10 mg/kg isoproterenol-induced lipolysis in WT versus Myeloid-KO mice; *n* = 8/group. (**C** and **D**) Blood glucose levels in response to 10 mg/kg isoproterenol-induced lipolysis in WT versus Endo-KO mice (*n* = 10/group) and in WT (*n* = 7) versus Adipo-KO (*n* = 10) mice. (**E** and **F**) Plasma FAPB4 and insulin responses in WT and *Fabp4^–/–^* mice injected with PBS or 7 μg of FABP4 prior to induction of lipolysis with 10 mg/kg isoproterenol; *n* = 8/group. All experiments were in male mice. ****P < 0.0001, ***P < 0.001, **P < 0.01, *P < 0.05 versus WT; °°°°*P* < 0.0001, °°°*P* < 0.001, °*P* < 0.05 versus Adipo-KO, by mixed-effects analysis, followed by Tukey’s (**A** and **F**) or Sidak’s (**C** and **E**) multiple-comparison test, or 2-way ANOVA followed by Tukey’s multiple-comparison test (**B** and **D**). ND, no signal detected.

In response to β-adrenergic induction of lipolysis, *Fabp4^–/–^* mice have markedly blunted insulin responses, suggesting that FABP4 deficiency alters islet β cell function during acute lipolysis (23). To examine whether this phenotype is due to absence of FABP4 in adipocytes or endothelial cells, we measured plasma insulin responses to isoproterenol-induced lipolysis. Surprisingly, despite the markedly blunted FABP4 induction in response to lipolysis, Adipo-KO mice did not show any significant reduction in insulin secretion (Figure 6A). There were also no changes in insulin responses in Myeloid-KO mice (Figure 6B). In contrast, peak insulin responses in Endo-KO mice were reduced by nearly 60% compared with WT mice, to a similar degree displayed by Total-KO mice. The reduced insulin secretion in Endo-KO mice occurred in spite of comparable absolute plasma FABP4 levels to Adipo-KO mice during lipolysis. Hence, these findings support that endothelial-source FABP4 is a key requirement for lipolysis-driven acute induction of insulin secretion.

Isoproterenol can induce increases in glucose, though the increases are small in comparison with the scale of induction of insulin secretion (22). However, we found that glucose levels in Endo-KO and Adipo-KO mice in response to isoproterenol did not differ from WT (Figure 6, C and D). These results are consistent with *Fabp4^–/–^* mice, where reduced lipolysis-induced insulin secretion is not accompanied by differences in glucose responses compared with WT mice (23) and, therefore, do not support a contribution from reduced glucose levels to the blunted insulin responses we observed here.

Another potential contribution to the decreased insulin responses in Endo-KO mice could be the low basal FABP4 levels in Endo-KO mice. To investigate this question, we administered *Fabp4^–/–^* mice with recombinant FABP4 by i.p. injection 30 minutes prior to induction of lipolysis. Despite reaching FABP4 levels comparable with WT controls (Figure 6E), insulin responses were not restored by FABP4 administration and even tended to be reduced further (Figure 6F) — an effect we have previously observed with in vivo FABP4 administration (24). These data suggest a few possibilities — that deficiency of FABP4 within endothelial cells may underlie the lack of lipolysis-associated insulin secretion; that endothelial-source FABP4 functions differently from adipocyte-source FABP4, a factor which is not captured by the use of recombinant protein; or that chronic low-circulating FABP4 levels in Endo-KO and Total-KO mice may alter β cell insulin responsiveness during lipolysis.

### Deficiency of endothelial FABP4 alters the insulin secretory response.

**Figure 7:**
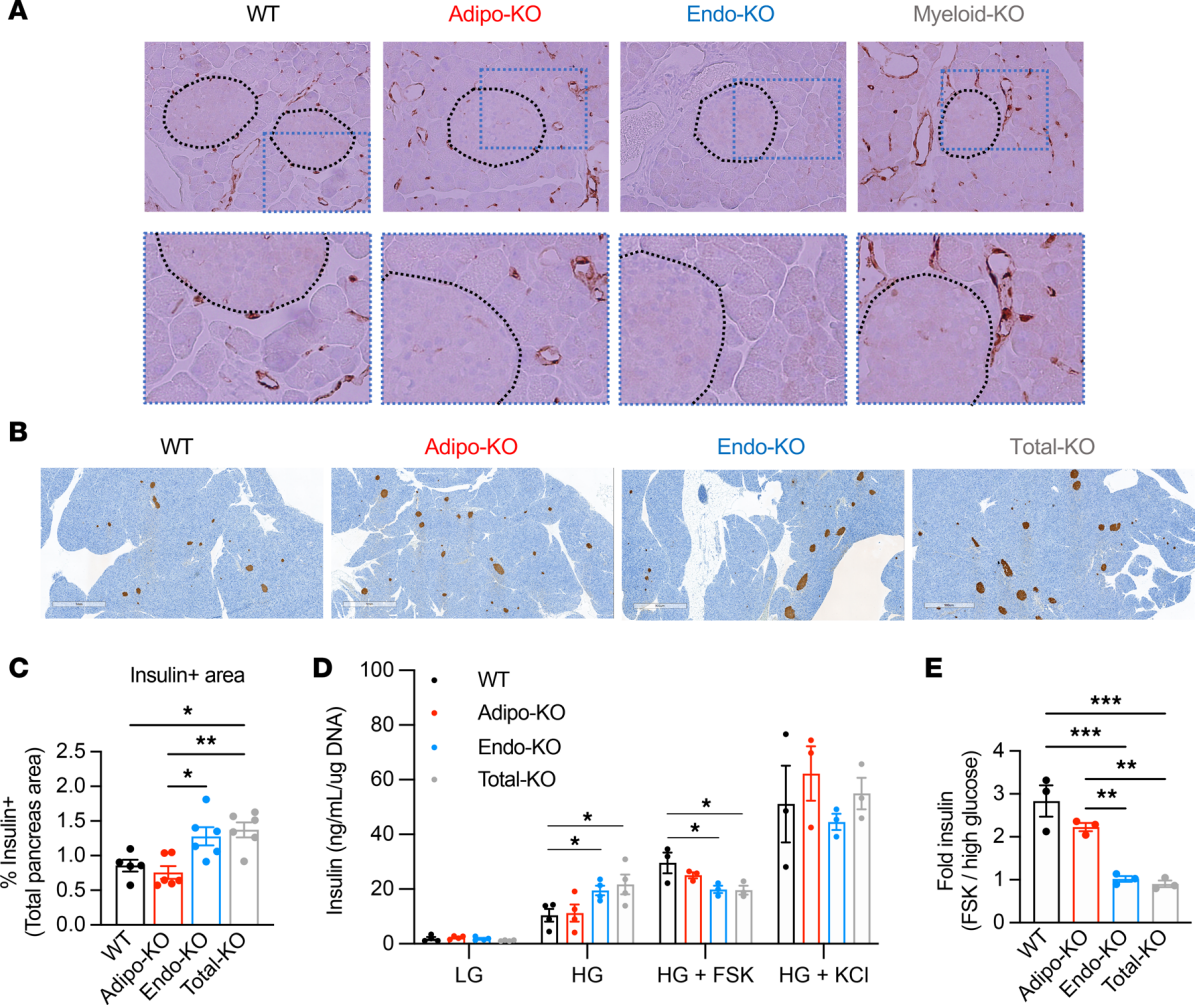
Pancreas endothelial cells express FABP4, and Endo-KO islets show altered regulation of insulin secretion. (**A**) Upper panel: Representative FABP4 immunostaining of pancreas of WT, Adipo-KO, Endo-KO, and Myeloid-KO mice. Islets are encircled by black dotted lines. Magnification, 400×. Lower panel: 2× enlargement of boxed area in upper panel. (**B**) Representative insulin immunostaining of pancreas from WT, Adipo-KO, Endo-KO, and Total-KO mice; magnification, 80×. (**C**) Quantification of insulin-positive area. Pool of 2 experiments. WT: *n* = 5; Adipo-KO, Endo-KO, Total-KO: *n* = 6/group. (**D**) Insulin secretion from isolated islets of WT, Adipo-KO, Endo-KO, and Total-KO mice in response to low glucose (LG, 2.8 mM), high glucose (HG, 16.7 mM), HG + forskolin (FSK, 10 μM), and HG + KCl (30 mM). Insulin secretion is normalized to cellular DNA. LG, HG: *n* = 4/group. HG + FSK or KCl: *n* = 3/group. (**E**) Fold-increase in insulin secretion induced by HG + FSK (10 μM) over HG from 7D. *P < 0.05, **P < 0.01, ***P < 0.001 by 1-way ANOVA followed by Tukey’s (**C** and **E**) or Dunnett’s (**D**) multiple-comparison test. HG Endo-KO is *P* < 0.05 by *t* test (**D**).

To examine the potential role for endothelial FABP4 in insulin secretion from islets, we performed immunostaining for FABP4 and insulin on pancreatic sections and examined the ex vivo insulin secretion phenotype of WT, Adipo-KO, and Endo-KO mouse islets. In our earlier studies, we demonstrated that FABP4 is not expressed in isolated islets or β cell lines (23, 24). Consistently, pancreata from WT and Adipo-KO mice showed minimal FABP4 immunoreactivity in islets (Figure 7A). However, there was FABP4 staining in the vasculature of the exocrine pancreas. This FABP4 signal was completely lost in the entire pancreas of Endo-KO mice but was observed in Myeloid-KO mice, suggesting that the FABP4^+^ cells were primarily endothelial cells. Recent evidence supports an integrated capillary network of bidirectional blood flow between the exocrine and endocrine pancreas (37). These data raise the possibility that FABP4 generated from endothelial cells may have the capacity to influence β cell function.

Despite the reduced lipolysis-induced insulin secretion in Endo-KO and Total-KO mice, staining of pancreatic sections revealed increases in insulin-positive area compared with WT and Adipo-KO mice (Figure 7, B and C), similar to our previous observation of increased β cell mass in FABP4-deficient mice (24). In contrast to the decreased in vivo insulin responses during lipolysis, primary islets isolated from Endo-KO and Total-KO mice showed increased GSIS compared with WT islets (Figure 7D). This was not due to a general increase in insulin secretion, since addition of the secretagogue, KCl, showed no difference in the maximal secretory capacity between all groups. These findings are supported by our previous results in *Fabp4^–/–^* mice, where the response to the insulin secretagogue L-arginine was intact (23) and islets from these animals exhibited a superior GSIS (24).

In contrast to high glucose (HG) alone, further induction of insulin secretion by increasing intracellular cAMP with FSK was reduced in Endo-KO and Total-KO islets compared with WT (Figure 7, D and E), mirroring the reduced insulin response to isoproterenol-induced lipolysis in vivo. We used FSK instead of isoproterenol because it has been shown that treatment of islets with doses of isoproterenol of 10 μM or higher can inhibit insulin secretion (38, 39). To confirm that the differences in in vivo insulin responses were not due to altered sensitivity of the islets to isoproterenol, we treated islets with 10 μM isoproterenol in the presence of HG. We chose this dose to mimic the expected concentration obtained with 10 mg/kg dosing in vivo. In total, 10 μM isoproterenol uniformly suppressed insulin secretion in WT, Adipo-KO, and Endo-KO islets (Supplemental Figure 10), confirming that the induction of insulin in vivo occurs in spite of a direct suppressive effect of isoproterenol on islets. Importantly, the reduced in vivo insulin responses in Endo-KO and Total-KO mice are not due to altered islet sensitivity to isoproterenol. Taken together, our data demonstrate that islets from Endo-KO and Total-KO mice show enhanced GSIS, but reduced cAMP-mediated insulin responses, from a pathway that is potentially independent of β-adrenergic signaling.

## Discussion

Since the discovery that adipocytes secrete FABP4 and that circulating FABP4 is elevated in obesity (10, 13), it has generally been presumed that adipocytes are the major source of plasma FABP4 and are responsible for its hormonal effects. Here, unexpectedly, we report that, in lean mice under basal unstimulated conditions, adipocytes only contribute ~11% of steady-state circulating FABP4; instead, endothelial cells are the predominant source, contributing to ~87% of baseline plasma FABP4. In contrast, in response to β-adrenergic–induced lipolysis, adipocytes are the major source of lipolysis-driven increases in circulating FABP4. Surprisingly, despite significantly diminished induction of FABP4, mice with adipocyte-specific FABP4 deficiency have essentially intact acute insulin responses to lipolysis. Conversely, mice lacking endothelial FABP4 have markedly diminished insulin responses, similar to whole-body *Fabp4^–/–^* mice, despite only having mildly reduced stimulation of FABP4 secretion, and absolute plasma FABP4 levels during lipolysis, similar to mice with adipocyte FABP4 deletion. We demonstrate for the first time to our knowledge that the endothelium is the major source of baseline circulating FABP4 and that endothelial FABP4 is a key requirement for lipolysis-induced insulin secretion.

Our data show that endothelial cells contribute significantly to circulating FABP4 in vivo, despite lower levels of expression and secretory capacity than adipocytes. As in adipocytes, secretion did not occur through the classical ER-Golgi pathway in the endothelial cells used in this study (13, 15), but unlike adipocytes, no FABP4 was detected in exosomes from endothelial cells. Another major difference, at least in vitro, is that endothelial FABP4 secretion appears to be constitutive, rather than inducible, as demonstrated by the lack of FABP4 response to β-adrenergic stimuli and increases in intracellular cAMP. Further studies are warranted to reach definitive conclusions and explore the differences between in vivo and in vitro responses.

The differential cellular sourcing of baseline versus induced FABP4 secretion may have significant implications for understanding the pathological versus physiological effects of circulating FABP4. It is well established that FABP4-deficient mice are protected against obesity-induced hyperinsulinemia, insulin resistance, and glucose intolerance (3, 5). It is also well established that basal lipolysis is constitutively increased in obesity (40); therefore, one possible mechanism for protection of FABP4-deficient mice from insulin resistance and its consequences may be decreased lipolysis-mediated insulin secretion, which is significantly reduced in these animals (23). Recent evidence supports a model in which prevention of hypersecretion of insulin and hyperinsulinemia may be critical for alleviation of insulin resistance and protecting β cell functionality and survival (41). Free fatty acids (FFAs) possess an important role in this regulatory circuit, as they are critical for glucose- and nonglucose-induced insulin secretion (42–44). FFA-mediated stimulation of insulin secretion occurs through activation of the fatty acid receptor FFAR1 on β cells (45–47). Here, whole-body and endothelial *Fabp4^–/–^* mice show markedly blunted insulin responses to β-adrenergic–induced lipolysis despite only minimal or no reductions in NEFA and glycerol responses. Interestingly, *Ffar1*-KO mice only show partially decreased insulin responses to β-adrenergic–induced lipolysis (48), indicating that signals in addition to FFAs are involved in lipolysis-mediated insulin secretion. Here, we show that FABP4 specifically produced from endothelial cells is a key requirement for lipolysis-mediated insulin secretion. Moreover, endothelial FABP4 promotion of lipolysis-mediated insulin secretion may be detrimental for β cell health and function, since hyperinsulinemia is a driver for the development of insulin resistance (49).

The small induction of FABP4 during lipolysis in mice with adipocyte deletion of FABP4 indicated secretion of FABP4 from endothelial cells. One possibility is that this small increase in endothelially derived FABP4 could be the driver of lipolysis-induced insulin secretion. Comparative analysis of FABP4 secreted from endothelial cells and adipocytes would be of interest to determine whether FABP4 from these 2 sources have different biochemical forms, such as different posttranslational modifications or cargo, that could enable distinct functions. Though coculture of endothelial cells with islets or treatment of islets with conditioned media from endothelial cells would enable the determination of whether endothelially derived FABP4 directly influences insulin secretion, these experiments were not feasible due to the very low amounts of FABP4 secreted from endothelial cells and incompatibility of endothelial culture media with islets.

Our data demonstrate that neither the total body, nor the endothelial-specific deletion of FABP4, results in defective insulin secretion or decreased islet insulin content. In fact, both islet insulin content and glucose-stimulated insulin responses to glucose were increased in these models, supporting the suppressive role of FABP4 on pancreatic β cell mass and the proper function of islets (24). The increased islet mass and changes in islet insulin secretion that persisted ex vivo suggests a potential developmental role for endothelial FABP4 in β cell proliferation and function. This could be due to an effect of chronic low levels of circulating FABP4 or altered endothelial function due to absence of intracellular FABP4. This is intriguing, given that *Fabp4^–/–^* mice show decreased endothelial responsiveness to the proliferative effects of vascular endothelial growth factor (VEGF) (50), and VEGF has an important role in pancreatic vascular development, which in turn influences β cell function (51). In contrast to the increased insulin content and increased responses to glucose, islets isolated from either of these models show a blunted insulin response to FSK stimulation, suggesting that absence of endothelial FABP4 decreases cAMP-mediated amplification of insulin secretion. The differential effect of endothelial FABP4 on glucose-induced and cAMP-enhanced insulin secretion may indicate a context-dependent role of endothelial FABP4 on β cell insulin secretion. We have recently reported that FABP4 in complex with the kinases NDPK and ADK, directly inhibits β cell glucose-induced insulin secretion (24). This is in agreement with the enhanced GSIS that we observe in Endo-KO and Total-KO islets and supports a hormonal role of FABP4 in insulin secretion. Further studies are warranted to determine whether FABP4, either alone or in complex, can have a differential effect on β cell cAMP-mediated insulin responses.

The effect of endothelial cells to secrete FABP4 and modulate insulin secretion supports a role for the endothelium as an endocrine organ and a potential key regulator of metabolism. There is growing evidence for an important role of the endothelium in regulating systemic metabolism, and endothelial dysfunction is now recognized as an important early event in the pathogenesis of obesity (29, 30). In future studies, it will be important to determine the effect of cellular sources of FABP4 on the pathogenesis of conditions such as cancer, obesity, and cardiovascular disease, where its circulating levels are regulated and known to be critical for systemic disease.

## Methods

### Generation of Fabp4-floxed mice and tissue-specific deletion models.

Studies were performed in humanized *Fabp4*-floxed mice on a C57BL/6 background generated for the Hotamışlıgil lab by GenOway. The *Fabp4* gene is located on chromosome 3 and consists of 4 exons encoding a 132–amino acid, 15 kDa protein. A targeting strategy was developed based on analysis of the *Fabp4* gene organization and the predicted functional protein structure. The murine *Fabp4* sequence was first humanized by introducing point mutations to convert the 11 amino acids in exons 2 and 3 that differ between mouse and human *FABP4* to human *FABP4* sequences (Supplemental Figure 1B). This will allow for future studies to develop targeting therapies against human FABP4. A *lox*P site was inserted within intron 1 upstream of exon 2 and a FRT-neomycin-FRT-*lox*P cassette was inserted at the 3′ end of exon 2 (Supplemental Figure 1C). The targeting construct was electroporated into C57BL/6 embryonic stem (ES) cells. The positive ES cell clones were injected into blastocysts of albino C57BL/6J mice (C57BL/6J-Tyrc^–2J^/J), which were implanted into pseudo-pregnant females. Highly chimeric male pups were bred to whole-body Flp recombinase–expressing mice to excise neomycin selection cassette. The resulting *Fabp4*-floxed mice were crossed to Adiponectin-Cre (B6.FVB-Tg[Adipoq-cre]1, gift from E.D. Rosen, Beth Israel Deaconess Medical Center, Boston, Massachusetts, USA) (52), Tek-Cre (B6.Cg-Tg[Tek-cre]1Ywa/J, The Jackson Laboratory, stock no. 008863) (53), LyzM-Cre (B6.129P2-*Lyz2^tm1(cre)Ifo^*/J, The Jackson Laboratory, stock no. 004781) (54), and CMV-Cre (B6.C-Tg[CMV-cre]1Cgn/J, The Jackson Laboratory, stock no. 006054) (55) mice, to generate mice with deletion of FABP4 in adipocytes (Adipo-KO), endothelial cells (Endo-KO), myeloid cells (Myeloid-KO), and the whole body (Total-KO), respectively (Supplemental Figure 1A). *Fabp4*–floxed CMV-Cre^+^ mice had germline deletion of *Fabp4*. In the majority of the Total-KO mice studied, the Cre recombinase was bred out by crossing to WT and retaining mice with only *Fabp4* deletion to cross with each other to generate homozygous *Fabp4*-floxed KO. In experiments where some Total-KO mice were Cre-positive, there were no differences from Cre^–^ mice. Initially, *Fabp4*-floxed mice were genotyped by in-house by PCR (fwd: 45835-Flp-HCA1, 5′ GAAGGTGAGGAACAAGGAGCTGATGC 3′; rev: 45836-Flp-HCA1, 5′ AGGTGGGCACGGTAATGTTATGGTG 3′, WT 333 bp, knock-in 453 bp). Subsequently, genotyping for *Fabp4*-floxed, *Fabp4*-floxed KO, and Cre recombinase was performed by real-time PCR with specific probes designed for each gene (Transnetyx). *Fabp4*-floxed mice expressing both Adiponectin and Tek-Cre were genotyped with Cre probes that were specific for Adiponectin-Cre and Tek-Cre. Except where indicated, male mice between 8 and 18 weeks of age were studied. Within each replicate experiment, mice were age matched. Mice were maintained on a 12-hour light/dark cycle in the Harvard T.H. Chan School of Public Health pathogen-free barrier facility with free access to water and chow diet (PicoLab Mouse Diet 20, no. 5058, LabDiet).

### Baseline blood glucose and plasma hormone measurements.

Mice were food deprived for 6 hours from 9 a.m. to 3 p.m. prior to sample collection. Blood was obtained from a tail nick in nonrestrained mice for measurement of glucose levels with a glucometer (Ascensia Diabetes Care) and collected into heparinized capillary tubes for measurement of plasma FABP4, FABP5, and insulin levels. FABP4 levels obtained from 6-hour food-deprived baseline lipolysis samples were also included in the analysis.

### In vivo and explant lipolysis.

Mice were food deprived from 9 a.m. to 3 p.m., and lipolysis was induced by injection of isoproterenol in PBS (10 mg/kg, i.p., Tocris Bioscience). Blood was collected from a tail nick into heparinized capillary tubes at baseline before injection and 15, 30, and 60 minutes after injection for measurement of plasma FABP4, insulin, glycerol, and NEFA levels.

To determine the role of circulating FABP4 in the lipolysis-induced insulin response, *Fabp4^–/–^* mice were administered FABP4 (7 μg, i.p., recombinant human FABP4, produced in-house) in PBS vehicle or PBS alone 30 minutes prior to induction of lipolysis with isoproterenol. Responses were compared with those of WT mice injected with PBS. The FABP4 injection experiment was performed in mice that were WT and KO for mouse *Fabp4* (3), on C57BL/6 background.

For explant lipolysis, PGWAT was collected from male mice and washed with DMEM (Thermo Fisher Scientific) containing 10% Cosmic calf serum (CCS-DMEM; Cytiva). Adipose tissue was minced into ~2 mm^3^ pieces; the pieces were washed with CCS-DMEM and then incubated for 1 hour in CCS-DMEM. For each mouse, 3 replicates of 8 pieces of adipose tissue were then transferred to 3 wells of a 6-well plate with CCS-DMEM and incubated for 1 hour at 37°C, after which media were collected for measurement of basal secretion of FABP4, glycerol, and NEFA. Next, lipolysis was induced by incubation in 20 μM FSK (Cayman Chemical) in CCS-DMEM for 1 hour at 37°C, after which the media were discarded. Explants were incubated for 1 hour at 37°C with fresh media containing 20 μM FSK, and media were collected for analysis of lipolysis-induced secretion. Secretion was normalized to weight of the explants.

### Adipocyte isolation.

PGWAT was collected and washed with PBS and transferred to ice-cold Krebs-Ringer HEPES (Boston Bioproducts), pH 7.4, containing 2% BSA and 2 mg/mL Type 1 collagenase (MilliporeSigma). The tissue was minced into ~1 mm^3^ pieces and digested at 37°C for 45 minutes, with shaking at 100 rpm. The digest was strained through a 100 μm filter and centrifuged at 600*g* for 10 minutes at room temperature. The floating adipocytes were transferred to PBS and centrifuged at 600*g* for 10 minutes at room temperature. After centrifugation, the adipocytes were removed and transferred to 5 mM EDTA in PBS containing 2% BSA and spun at 600*g* for 1 minute at room temperature. The adipocytes were transferred to a 1.5 mL tube, centrifuged again at 600*g* for 1 minute at room temperature, and any remaining liquid was removed. Equal volume of 2× RIPA buffer (Cell Signaling Technology) was added to the adipocytes for protein isolation.

### Primary endothelial cell isolation and culture.

Endothelial cells were isolated from liver alone (20-week-old mice) or liver, heart, and lungs, with or without spleens (13- to 14-week-old mice). Mice were deeply anesthetized with 300 mg/kg ketamine and 30 mg/kg xylazine and transcardially perfused with PBS. Tissues were removed and placed in DMEM with 1% penicillin-streptomycin (Lonza). Each tissue was minced to ~1–2 mm^3^ pieces in digestion buffer consisting of 2 mg/mL Type 1 collagenase in 1% BSA in PBS with 1 mM CaCl_2_ and 1 mM MgCl_2_. The minced tissues from each organ were combined and digested at 37°C, for 1 hour, with shaking at 100 rpm. The digests were then strained with a 70 μm filter and spun at 1,300 rpm for 5 minutes at 4°C. The cell pellets were washed twice with 0.1% BSA in PBS and centrifuged at 250*g* for 5 minutes at 4°C between washes. After washing, the cells were suspended in 1 mL of endothelial growth medium (ATCC, Vascular Cell Basal Medium supplemented with Endothelial Cell Growth Kit-VEGF) and counted. In total, 2 × 10^7^ cells were incubated with 20 μL of CD31-coated microbeads (Miltenyi Biotec, 130-097-418) in 180 μL of MACS separation buffer (Miltenyi Biotec) for isolation of endothelial cells, according to the manufacturer’s instructions. The isolated CD31^+^ cells were used for cell culture, collected for FACS analysis, or lysed in hypertonic lysis buffer (150 mM NaCl, 50 mM EDTA, 100 mM Triton-X-100) for protein analysis. For determination of FABP4 and FABP5 secretion, cells were plated on 0.1% gelatin-coated plates with endothelial growth media. After 12 hours, media were collected and cells were lysed for subsequent analyses.

### FACS analysis.

For FACS analysis, CD31^+^ cells isolated from liver were suspended in 100 μL of MACS separation buffer per 1 × 10^6^ cells. Cells were stained with 2 μL of CD31-FITC (Miltenyi Biotec, 130-102-970, clone 390) and 2 μL of anti–F4/80-APC (Miltenyi Biotec, 130-102-942, clone REA126) in the dark at 4°C for 10 minutes before being washed with 2 mL of MACS separation buffer and centrifuged at 300*g* at 4°C for 10 minutes. Supernatant was removed, and the pellet was resuspended in MACS buffer and analyzed by flow-cytometer (BD FACSCalibur Flow Cytometer, BD Biosciences).

### HUVEC cell culture experiments.

Primary HUVECs (ATCC, passage 1) were cultured in endothelial growth medium (ATCC) in 0.1% gelatin-coated (Stemcell Technologies) tissue culture flasks. Passages 3–6 were used for experiments. HUVECs were seeded onto 12-well collagen-coated tissue culture plates (Corning) at a density of 1 × 10^5^ cells/well (day 0). The cells reached 100% confluence around day 3 and cobblestone stage around day 7. Intracellular FABP4 protein expression and secretion were measured from days 4 through 14 of culture. FABP4 secretory responses to the following agents were determined on days 11 or 12. ER-Golgi pathway secretion inhibitors included the following: brefeldin A (Tocris Bioscience) and monensin sodium salt (Tocris Bioscience). Lysosomal secretion inhibitors included the following: chloroquine (N4-[7Chloro-4-quinolinyl]-N1,N1-dimethyl-1,4-pentanediamine diphosphate salt, MilliporeSigma) and ammonium chloride (Boston Bioproducts). Induction of intracellular calcium utilized histamine dihydrochloride (Tocris Bioscience). Lipolytic stimuli included FSK (Cayman Chemical), 3-isobutyl-1-methylxantine (IBMX, MilliporeSigma), isoproterenol hydrochloride (Tocris Bioscience), and CL-316,243 disodium salt (Tocris Bioscience). Dose responses to FSK were compared with those of 3T3-L1 differentiated mouse adipocytes (ZenBio), cultured as previously described (14). For treatment, cobblestone HUVECs were washed twice with PBS and incubated in fresh vascular cell basal medium with treatment reagents (as specified in Figure 5 and Supplemental Table 1). After treatment, media were collected and spun at 500*g* for 15 minutes at 4°C. The supernatant was used as conditioned medium. The cells were washed twice with cold PBS and lysed for subsequent protein analyses.

### HUVEC exosome isolation.

Passage 2 HUVECs were cultured in growth media in ten 10 cm dishes until they reached the cobblestone stage. For isolation of secreted exosomes, plates were washed twice with PBS and incubated with 6.5 mL basal medium for 24 hours. Medium was pooled and centrifuged for 10 minutes at 1,000*g* at 4°C. The supernatant was passed through an 0.8 μm filter to remove cellular debris and larger extracellular vesicles, and it was split into three 20 mL samples for exosome isolation and one ~10 mL sample that didn’t undergo further processing. The cells were washed with PBS and lysed in NP-40 lysis buffer (Thermo Fisher Scientific), and the lysates were pooled together. The 20 mL conditioned medium samples were concentrated through 3 kDa ultrafiltration units (Thermo Fisher Scientific) to remove metabolites and degraded proteins. The media were further concentrated through 100 kDa ultrafiltration units (MilliporeSigma) to isolate the > 100 kDa exosomes and larger proteins. The > 100 kDa media then underwent size separation through a Sephacryl S-500 HR column (MilliporeSigma), and fractions were collected. To determine which fractions contained exosomes, aliquots of the fractions were treated with PKH26 (MilliporeSigma), a fluorescent dye that binds to lipid membranes. The exosome fractions underwent Western blotting using antibodies against FABP4 (HRP-tagged clone 351.4.5E1.H3, made for the Hotamışlıgil laboratory by the Dana Farber Antibody Core, as previously described; ref. 20), the exosome markers, CD63 (System Biosciences, EXOAB-CD63-1) and ALiX (BioLegend, 634502), and the double-membrane protein, β-actin (Abcam, ab8224). HUVEC media, cell lysate, and exosome FABP4 levels were also determined by ELISA, as described below.

### Peritoneal macrophage collection.

Mice were injected with thioglycolate (1 mL, i.p., Becton Dickinson). Four days later, mice were euthanized and 5 mL of PBS was injected into the peritoneum. The peritoneal fluid containing macrophages was collected and spun down to pellet the cells. The cell pellet was lysed with 200 μL hypertonic lysis buffer. The resulting cell lysate was assayed for FABP4 levels by ELISA, as described below.

### Islet isolation and insulin secretion.

Primary mouse islet isolation and insulin secretion assays were performed as previously described (24). Briefly, 20 islets were hand picked, washed twice with Krebs-Ringer Buffer (KRB) without glucose, and preincubated in low glucose (LG; 2.8 mM) KRB for 1 hour at 37°C. KRB was then removed, and islets underwent successive incubations for 20 minutes at 37°C with 250 μL of fresh KRB containing LG, followed by HG (16.7 mM), HG with FSK (10 μM) or HG with isoproterenol (10μM), and finally HG with KCl (30mM), to determine maximal insulin secretory capacity. At the end of each incubation, supernatant was collected for analysis of insulin levels. Following the stimulation protocol, cells were lysed in 100 μL acid ethanol (70% ethanol, 1% HCl in water) and stored at 4°C overnight. Samples were dried with a SpeedVac and resuspended in 60 μL ultrapure water. DNA was quantified by Nanodrop (Thermo Fisher Scientific). Insulin was quantified using insulin HTRF (Cisbio) and normalized to DNA content.

### Plasma and media assays.

Plasma and media human FABP4 were measured by in-house FABP4 ELISA or a commercially available assay (Biovendor). For in-house FABP4 ELISA, we used anti-FABP4 antibodies produced for the Hotamışlıgil lab by the Dana Farber Antibody Core, as described previously (20) (clone 351.4.2E12.H1.F12 for capture, HRP-tagged clone 351.4.5E1.H3 for detection), and recombinant human FABP4 as a standard (R&D Systems). Plasma mouse FABP4, FABP5, insulin, and media vWF and endothelin-1 (ET-1) levels were determined by commercially available ELISA (mouse FABP4, Biovendor; FABP5, CircuLex; Insulin, Alpco or Crystal Chem; human vWF, R&D Systems; human ET-1, R&D Systems). Plasma and media NEFA, glycerol, and LDH were measured by enzymatic colorimetric assays (NEFA, FujiFilm; Glycerol, MilliporeSigma; LDH, Takara Bio).

### Protein extraction, SDS PAGE, and Western blotting.

Tissues were collected from 19- to 26-week-old mice after 6-hour daytime food withdrawal. Western blots for FABP4 and FABP5 were performed in protein lysates from whole tissue or isolated cells. To clear tissues of circulating FABP4 and FABP5, mice were transcardially perfused with saline prior to tissue collection. Whole tissues were lysed in ice-cold RIPA buffer containing 2 mM activated Na_3_VO_4_ (New England Biolabs) and 1% protease inhibitor cocktail (MilliporeSigma). Lysates were assayed for protein concentration by bicinchoninic assay (Thermo Fisher Scientific), diluted with 6× Laemmli buffer (Morganville Scientific), and boiled for 5 minutes at 95°C before being subjected to SDS-PAGE using 15% (made in-house) or 4%–20% (Bio-Rad) gels. Protein was transferred to PVDF membranes (Bio-Rad), which were then blocked for at least 1 hour in TBS-T with 5% protease-free BSA (MilliporeSigma). FABP4 protein was detected using HRP-tagged anti-FABP4 antibody (clone 351.4.5E1.H3, generated for Hotamışlıgil lab by Dana Farber Antibody Core; ref. 20). FABP5 was detected with rabbit anti-FABP5 antibody (Cell Signaling Technology, 39926S) followed by HRP-tagged anti-rabbit secondary antibody (Cell Signaling Technology, 7074). Membranes were incubated with HRP-tagged anti–β-tubulin antibody (Abcam, ab21058; Cell Signaling Technology, 5346) to measure β-tubulin as a loading control. HRP signal was detected with chemiluminescent substrate (Thermo Fisher Scientific). Antibodies have been validated with protein lysates from *Fabp4^–/–^* and *Fabp5*^–/–^ mice as negative controls and recombinant FABP4 and FABP5 as positive controls. The FABP5 was hexahistidine tagged (Cayman, 10007443) and was 19.3 kDa instead of the 15 kDa size of native FABP5.

### IHC.

Liver, adipose tissues, and pancreas were fixed in 10% zinc formalin overnight at room temperature and then transferred to 70% ethanol. Tissues were embedded in paraffin and sectioned at the Dana Farber Rodent Histopathology Core. Slides were deparaffinized and rehydrated; then, heat-induced epitope retrieval was performed by boiling in 10 mM citrate buffer, pH 6.0, for 15 minutes, followed by washing with deionized water. For pancreas, FABP4 immunostaining endogenous peroxidase activity was blocked by incubating for 5 minutes with 3% hydrogen peroxide in deionized water. Slides were washed, then incubated with serum-free protein block for 5 minutes (Leica Biosysems), followed by incubation with rabbit anti-FABP4 polyclonal antibody (1:800; MilliporeSigma, HPA002118) in antibody diluent (Agilent) overnight at 4°C. Slides were washed and incubated with anti–rabbit poly-HRP IgG secondary antibody (Leica Biosystems, RE7200-CE) for 30 minutes at room temperature. They were then washed again, and peroxidase activity was developed with 3,3′-diaminobenzidine chromogen and peroxidase substrate, and they were counterstained with hematoxylin.

For pancreas islet insulin immunostaining, 3 serial sections per pancreas 250 μm apart were processed. Immunostaining was performed as described previously (24). Images of each section were acquired using Aperio ImageScope at 20× magnification. Images were further digitally magnified by 4×, and β cell area was calculated by using positive pixel count analysis (Aperio ImageScope). Islet number and size were determined by manually circling insulin-positive clusters in the Aperio ImageScope software; this process was performed blinded to the sample identifications.

### Statistics.

Statistical analysis was performed using GraphPad Prism version 9.4.0 for MacOS, GraphPad Software, San Diego, California, USA (www.graphpad.com). Data with more than 2 groups were analyzed by 1-way ANOVA, followed by Tukey’s or Sidak’s multiple-comparison test or Dunnett’s multiple-comparison test, in cases where comparisons were made to only the WT control group or a baseline value. For comparison of 2 groups, data were analyzed by 2-tailed unpaired Student’s *t* test. Data with 2 or more groups plotted over multiple time points were analyzed by 2-way repeated-measures ANOVA or mixed-effects model, if data points were missing, followed by Tukey’s or Sidak’s multiple-comparison test. A *P* value less than 0.05 was considered significant. Values were removed from analysis if they were further than 2 SDs from the mean or determined to be outliers by the ROUT method. Statistical methods are indicated in the figure legends. All data are presented as mean ± SEM.

### Study approval.

All mouse studies were approved by the Harvard Medical Area Standing Committee on Animals.

### Data availability.

All data for this publication are included in the main article, Supplemental Data and unedited gel image files, and Supporting Data file.

## Author contributions

KEI designed and performed the experiments and assays, analyzed and interpreted the data, and prepared the manuscript. MFB helped to initiate the project and performed the first phenotyping of the Adipo-KO mice. AL, KJP, CDG, MXC, JKR, and GYL assisted with in vivo experiments. KJP and AL designed and performed the primary endothelial experiments. KJP designed and performed in vitro islet experiments. GYL designed and performed the HUVEC and 3T3-L1 experiments. ZBW performed the HUVEC exosome experiments and 3T3-L1 culture. AL, CDG, MXC, and JKR performed tissue and plasma assays. KJP, AL, and GYL analyzed the data and revised the manuscript. GSH conceived, supervised, and supported the project; designed experiments and controls; interpreted results; and revised the manuscript.

## Supplementary Material

Supplemental data

Supporting data values

## Figures and Tables

**Table 1:**
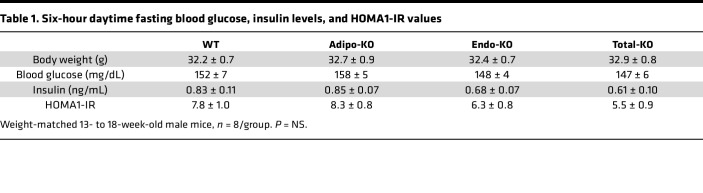
Six-hour daytime fasting blood glucose, insulin levels, and HOMA1-IR values

**Table 2:**
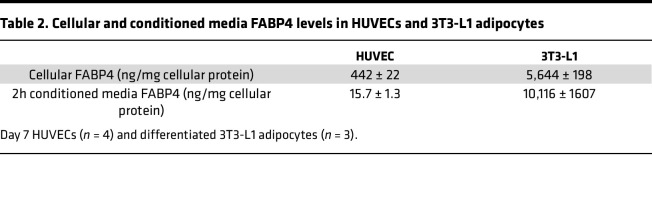
Cellular and conditioned media FABP4 levels in HUVECs and 3T3-L1 adipocytes

## Version 1 06/06/2023

In-Press Preview

## Version 2 07/24/2023

Electronic publication
