# Supplementary material for: Endothelial-derived FABP4 constitutes the majority of basal circulating hormone and regulates lipolysis-driven insulin secretion
Source: JCI Insight. 2023 Jul 24;8(14):e164642. doi: 10.1172/jci.insight.164642 (PMC10443803; doi:10.1172/jci.insight.164642)
Supplement: Supplemental data [file jciinsight-8-164642-s215.pdf]

Supplemental Data

Endothelial FABP4 constitutes the majority of basal circulating hormone levels and regulates lipolysis-driven insulin secretion

Karen E. Inouye, Kacey J. Prentice, Alexandra Lee, Zeqiu B. Wang, Carla Dominguez-Gonzalez, Mu Xian Chen, Jillian K. Riveros, M. Furkan Burak, Grace Y. Lee, Gökhan S. Hotamışlıgil

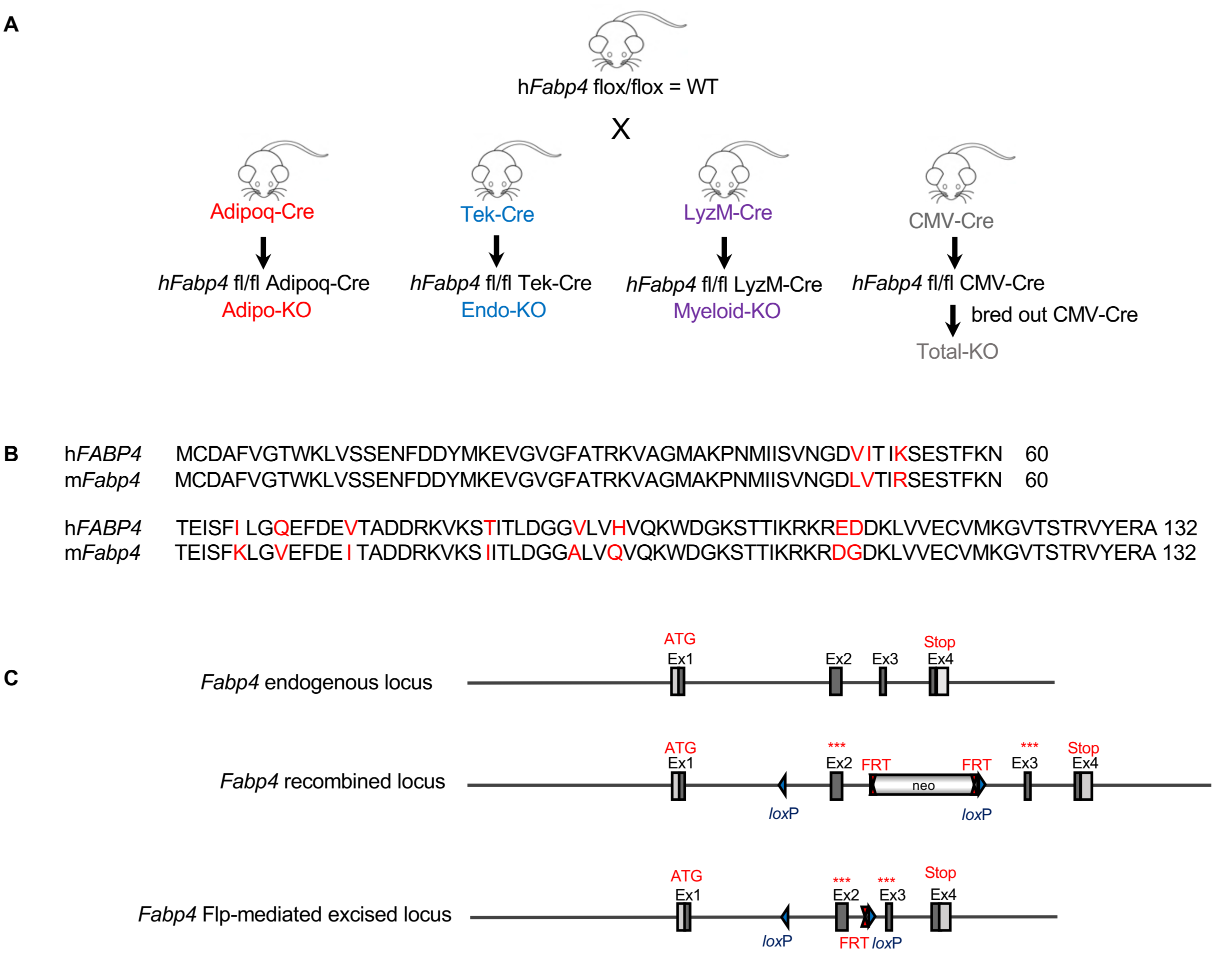

Supplementary Figure 1. Breeding scheme and construct design for tissue-specific FABP4 deletion mice. (**A**) Mice with floxed humanized *Fabp4* (*hFabp4* flox/flox) were crossed with Adiponectin-Cre, Tek-Cre, LyzM-Cre, or CMV-Cre mice to generate mice with deletion of FABP4 in adipocytes (Adipo-KO), endothelial cells (Endo-KO), myeloid cells (Myeloid-KO), or the whole body (Total-KO). Since crossing with CMV-Cre produces germline deletion of *hFabp4*, CMV-Cre was bred out of the majority of the Total-KO mice used for these studies. *hFabp4* flox mice are referred to as WT. WT mice used in this study are littermates of Adipo and Endo-KO mice that did not express Cre recombinase. (**B**) The mouse *Fabp4* gene was first humanized by substituting the 11 amino acids differing between mouse and human *FABP4* located in exons 2 and 3. (**C**) Exon 2 was flanked at the 3' end by a FRT-neomycin-FRT-*lox*P cassette and by a single *lox*P site at the 5' end. The distal *lox*P site was positioned upstream of exon 2 within the intron 1 sequences. The model was generated by homologous recombination in embryonic stem cells. The FRT-flanked selection cassette was removed in vivo by crossing with Flp-recombinase-expressing mice. Dark gray rectangles represent *Fabp4* coding sequences. Light gray rectangles indicate non-coding exon portions. Solid lines represent chromosome sequences. The 11 murine/human amino acid substitutions are represented by stars.

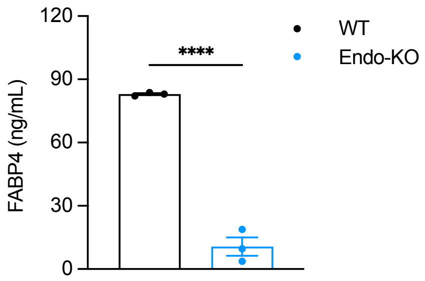

Supplementary Figure 2. **FABP4 is deleted from macrophages of Endo-KO mice.** Peritoneal macrophage lysate FABP4 levels from WT and Endo-KO mice. ****P < 0.0001 by unpaired t-test.

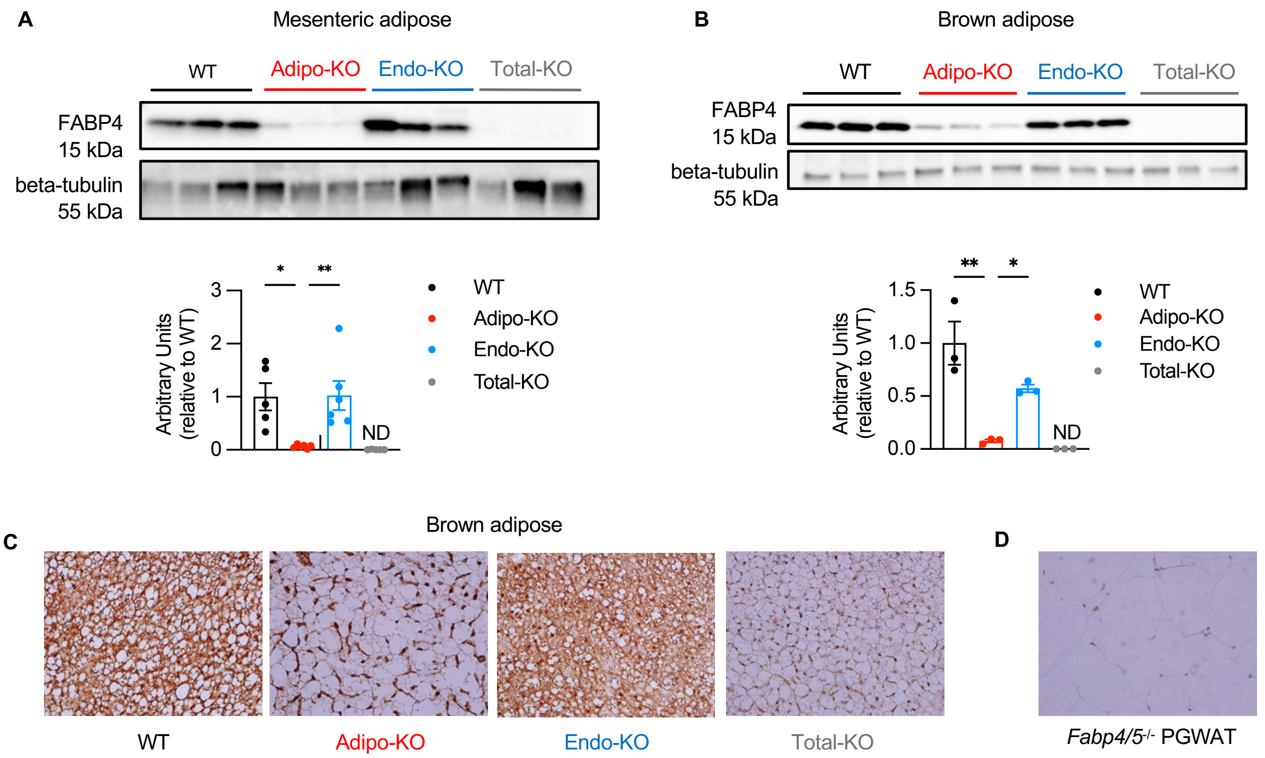

Supplementary Figure 3. Validation of tissue-specific deletion of FABP4 from adipocytes. (**A**) Representative immunoblots (n=2) and quantification of FABP4 protein relative to beta-tubulin loading control in mesenteric adipose tissue of WT, Adipo-KO, Endo-KO, and Total-KO mice. WT, Total-KO: n=5/group; Adipo-KO, Endo-KO: n=6/group. (**B**) Immunoblot and quantification of FABP4 expression relative to beta-tubulin loading control in brown adipose tissue of WT, Adipo-KO, Endo-KO, and Total-KO mice. n=3/group. (**C**) Representative FABP4 immunostaining in brown adipose tissue from WT, Adipo-KO, Endo-KO, and Total-KO mice. 40X magnification. (**D**) Representative FABP4 immunostaining in perigonadal adipose tissue (PGWAT) from *Fabp4/5*$^{-/-}$ mice. 40X magnification. *P < 0.05, **P < 0.01 by 1-way ANOVA, followed by Tukey's multiple comparison test. ND: No signal detected.

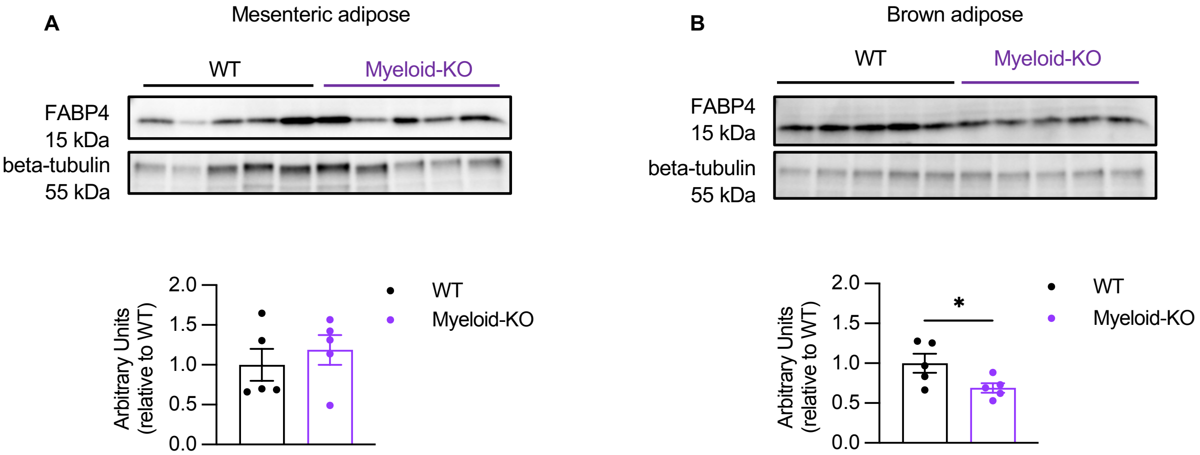

Supplementary Figure 4. FABP4 is decreased in brown adipose tissue of Myeloid-KO mice. Immunoblots and quantification of FABP4 protein relative to beta-tubulin loading control in (**A**) mesenteric and (**B**) brown adipose tissue of WT and Myeloid-KO mice. n=5/group. *P < 0.05 by unpaired t-test.

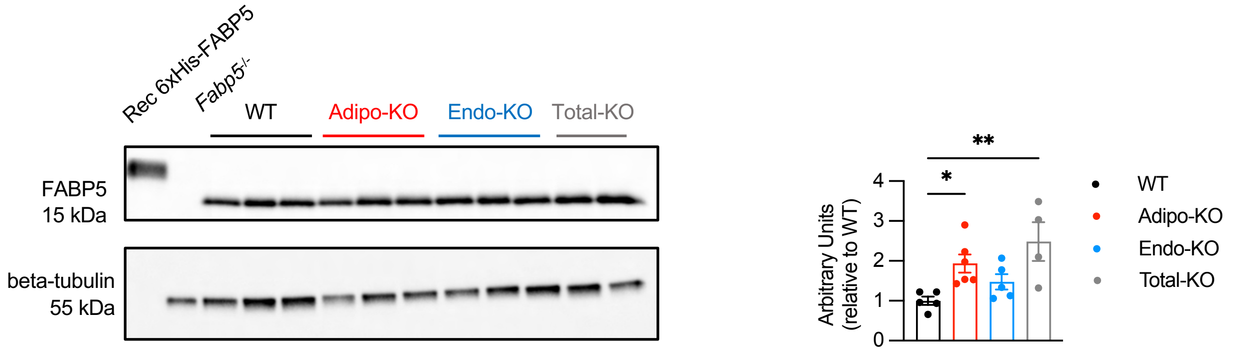

Supplementary Figure 5. FABP5 is increased in perirenal adipose of Total-KO mice. Representative immunoblot (n=2) and quantification of perirenal adipose FABP5 protein relative to beta-tubulin loading control in WT, Adipo-KO, Endo-KO, and Total-KO mice. WT, Adipo-KO, Endo-KO: n=5/group. Total-KO: n=4. Recombinant hexahistidine-tagged FABP5, which travels at 19 kDa, was used as a positive control. *Fabp5*$^{-/-}$ adipose lysate was a negative control. *$P < 0.05$, **$P < 0.01$ by 1-way ANOVA, followed by Dunnett's multiple comparison test.

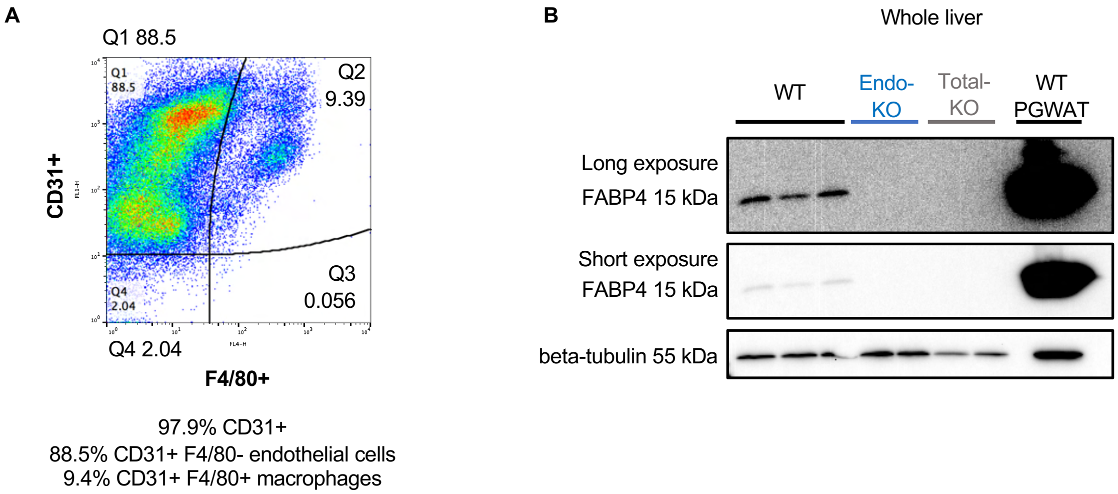

Supplementary Figure 6. 88.5% of liver CD31-isolated cells are endothelial cells, and FABP4 protein signal in liver comes from endothelial cells. (**A**) FACS analysis of WT liver endothelial cells isolated with anti-CD31 antibody-coated beads, and co-stained with anti-CD31-FITC and anti-F4/80-APC to determine the percentage of F4/80-positive macrophages within the CD31 population. From n=1 WT mouse. (**B**) Immunoblots of FABP4 protein and beta-tubulin loading control in liver lysates from WT, Endo-KO, and Total-KO mice. WT: n=3. Endo-KO, Total-KO: n=2/group. Lysates from perigonadal adipose (PGWAT) of WT mice included as a positive control.

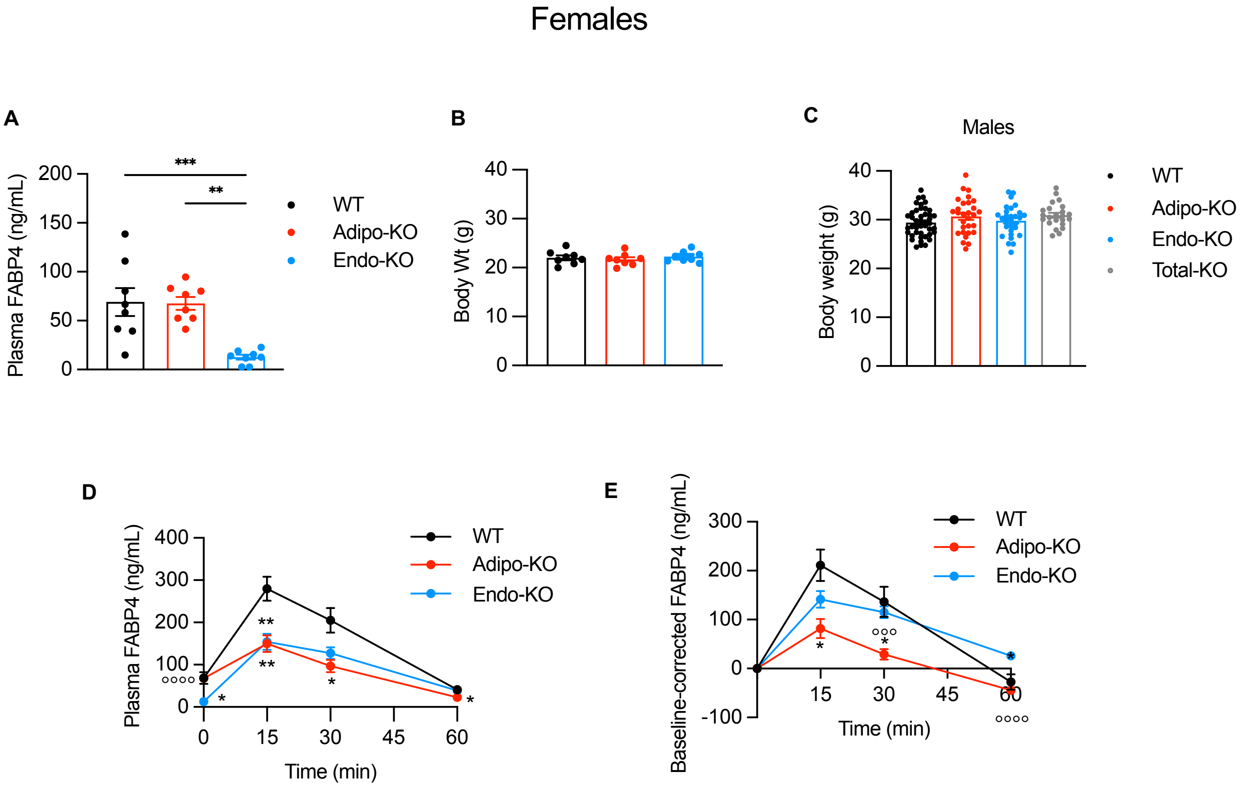

Supplementary Figure 7. Baseline and lipolysis-source FABP4 in females is similar to males. (**A**) 6h daytime fasted plasma FABP4 levels and (**B**) body weights of lean ~12 week-old female WT, Adipo-KO, Endo-KO, and Total-KO mice from S7D lipolysis experiment. n=8/group. (**C**) Body weights of males from Figure 3A 6h fasting FABP4 levels. WT (n=43), Adipo-KO (n=29), Endo-KO (n=29), and Total-KO (n=23) (**D**) Absolute and (**E**) baseline-corrected plasma FABP4 responses to 10mg/kg isoproterenol-induced lipolysis in female WT, Adipo-KO, and Endo-KO mice. n=8/group. ****P* < 0.001, ***P* < 0.01, **P* < 0.05 vs. WT; °°°°*P* < 0.0001, °°°*P* < 0.001 vs. Endo-KO, by 1-way ANOVA (**A** - **C**) or mixed-effects model (**D, E**), followed by Tukey's multiple comparison test.

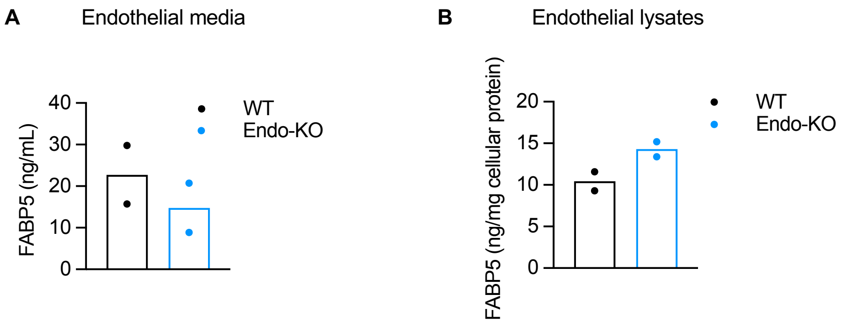

Supplementary Figure 8. FABP5 levels are not changed in Endo-KO mice. (**A**) FABP5 levels in overnight conditioned media and (**B**) lysates of CD31-isolated endothelial cells from liver, spleen, heart, and lung of WT and Endo-KO mice. Lysates were from freshly isolated cells.

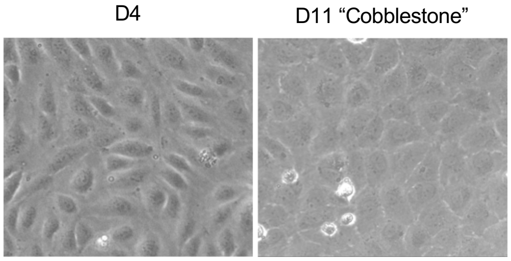

Supplementary Figure 9. Light microscopy image of HUVECs at days 4 and 11 post-seeding. Day 11 HUVECs demonstrate “cobblestone” phenotype. 10X magnification.

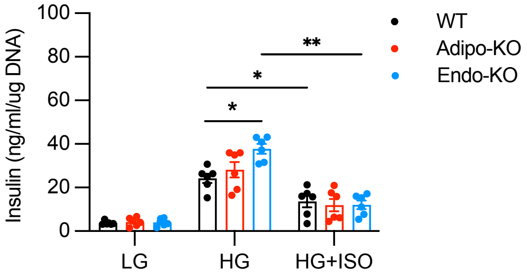

Supplementary Figure 10. Isoproterenol inhibits islet insulin secretion. Insulin secretion from isolated islets of WT, Adipo-KO, and Endo-KO mice in response to low glucose (LG, 2.8mM), high glucose (HG, 16.7mM), high glucose + isoproterenol (ISO, 10μM). Insulin secretion is normalized to cellular DNA. n=3 males, n=3 females per group. **P* < 0.05, ***P* < 0.005 by 1-way ANOVA, followed by Tukey's multiple comparison test.

Supplementary Table 1: Agents that did not affect HUVEC FABP4 secretion

		**Reagent**
**Secretory Mechanisms**	**ER-Golgi pathway inhibition**	Brefeldin A, monensin (Up to 20uM)
	**Lysosomal pathway inhibition**	Chloroquine (Up to 50uM)<br>NH_4Cl (Up to 50mM)
	**Increased intracellular Ca++**	Histamine (Up to 25uM)
**Stimuli**	**Lipolytic/Adrenergic**	FSK (Up to 25uM), IBMX (1mM)<br>CL-316,243 (Up to 20uM)<br>Isoproterenol (Up to 50uM)

Day 12 HUVECs were treated for 2 hours with agents known to target secretory mechanisms in adipocytes and endothelial cells, and stimulate adipocyte FABP4 secretion.
